# *Gl*PRMT5 inhibits *Gl*PP2C1 via symmetric dimethylation and regulates the biosynthesis of secondary metabolites in *Ganoderma lucidum*

**DOI:** 10.1038/s42003-024-05942-y

**Published:** 2024-02-28

**Authors:** Zi Wang, Hao Qiu, Yefan Li, Mingwen Zhao, Rui Liu

**Affiliations:** Key Laboratory of Agricultural Environmental Microbiology, Ministry of Agriculture and Rural Affairs; Microbiology Department, College of Life Sciences, Nanjing Agricultural University, Nanjing, 210095 Jiangsu P.R. China

**Keywords:** Fungal physiology, Fungal genetics

## Abstract

PRMT5, a type II arginine methyltransferase, is involved in transcriptional regulation, RNA processing and other biological processes and signal transduction. Secondary metabolites are vital pharmacological compounds in *Ganoderma lucidum*, and their content is an important indicator for evaluating the quality of *G. lucidum*. Here, we found that *Gl*PRMT5 negatively regulates the biosynthesis of secondary metabolites. In further in-depth research, *Gl*PP2C1 (a type 2C protein phosphatase) was identified out as an interacting protein of *Gl*PRMT5 by immunoprecipitation-mass spectrometry (IP-MS). Further mass spectrometry detection revealed that *Gl*PRMT5 symmetrically dimethylates the arginine 99 (R99) and arginine 493 (R493) residues of *Gl*PP2C1 to weaken its activity. The symmetrical dimethylation modification of the R99 residue is the key to affecting *Gl*PP2C1 activity. Symmetrical demethylation-modified *Gl*PP2C1 does not affect the interaction with *Gl*PRMT5. In addition, silencing *GlPP2C1* clearly reduced GA content, indicating that *Gl*PP2C1 positively regulates the biosynthesis of secondary metabolites in *G. lucidum*. In summary, this study reveals the molecular mechanism by which *Gl*PRMT5 regulates secondary metabolites, and these studies provide further insights into the target proteins of *Gl*PRMT5 and symmetric dimethylation sites. Furthermore, these studies provide a basis for the mutual regulation between different epigenetic modifications.

## Abstract (web summary)

The authors uncover the molecular mechanisms through which the arginine methyltransferase PRMT5 regulates the biosynthesis of secondary metabolites in the fungus Ganoderma lucidum.

## Introduction

Protein arginine methyltransferase 5 (PRMT5), a type II arginine methyltransferase member with clear functions, mainly participates in transcription regulation, RNA processing and other biological processes and signal transduction by symmetrically dimethylating target proteins^1^. For instance, *At*PRMT5 mutants exhibit distinct phenotypic changes including decreased plant height, altered leaf morphology, and degeneration of floral organs in *Arabidopsis*^2^. Moreover, PRMT5 contributes to secondary metabolite pigment biosynthesis in *Penicillium expansum*^3^. Studies have found that the main functional mode of PRMT5 is to directly methylate histones and nonhistones, thereby affecting a variety of physiological processes. Most studies have focused on the function of PRMT5 in affecting histone methylation^4–6^, and there are fewer studies on nonhistone methylation. As research progressed, some nonhistone proteins (enzymes or functional proteins) modified via PRMT5-mediated symmetric dimethylation were found to be involved in signal transduction pathways. For example, PRMT5-mediated symmetric dimethylation of the VP1 arginine (R) 426 residue impaired apparently VP1 polymerase activity, resulting in impaired viral replication^7^. PRMT5-mediated symmetric dimethylation of the R124 residue in cyclic GMP-AMP synthetase (cGAS) blocks the DNA-binding ability of cGAS to attenuate cGAS-mediated antiviral immune responses^8^. Crucially, there are few reports on PRMT5 target proteins in fungi via methylation modification. Exploring the downstream proteins modified by PRMT5 through nonhistone methylation will help elucidate the function of PRMT5 and fill gaps in this field.

Type 2C protein phosphatases (PP2Cs) are a large family of Mg^2+^/Mn^2+^-dependent phosphatases^9^. Published research clearly shows that PP2C plays a crucial role in many signal transduction pathways and physiological functions and responds to various environmental stresses. For example, overexpressing *AtPP2CF1* enhanced plant biomass production by activating cell proliferation and expansion to accelerate inflorescence stem growth in *Arabidopsis*^10^. Overexpression of *OsPP108* (a PP2C from rice) resulted in improved tolerance of transgenic *Arabidopsis* plants with better physiological parameters for fresh weight, chlorophyll content and photosynthetic potential under salt, mannitol and drought stress^11^. In addition, research on protein phosphatases of the PP2C family in microorganisms has been reported successively. For instance, *Saccharomyces cerevisiae* cells lacking Ptc1 (a type 2C protein phosphatase) showed high sensitivity to exogenous stresses such as high pH, LiCl, CaCl_2_, ZnCl_2_, CFW, caffeine, and rapamycin^12^. *Fusarium oxysporum* Ptc6 (type 2C protein phosphatase) mutants showed increased sensitivity to membrane (SDS), cell wall (CFW), and oxidative (menadione) stress compounds^13^. Most studies have reported the downstream mechanism of PP2C as a phosphatase regulator^14,15^. In addition, the upstream mechanism of PP2C regulation has also been reported. On the one hand, the expression of *PP2C* is inhibited at the transcriptional level to inhibit its activity. As an illustration, H_2_O_2_ produced by the ABA-induced NADPH oxidase RbohB/E inhibits PP45 (a type 2C protein phosphatase in rice) activity by inhibiting the expression of *PP45*^16^. *Os*bZIP12/*Os*ABF1 and *Os*bZIP46/*Os*ABF2 (the bZIP family transcription factors in rice) directly bind to the *Os*PP2C09 (a type 2C protein phosphatase in rice) promoter and mediate rapid induction of its expression after exogenous ABA treatment^17^. On the other hand, posttranslational modifications affect PP2C activity. As evidence, ABA affects PP45 activity by oxidizing cysteine (Cys-350 and Cys-428) residues to form intermolecular dimers of PP45^16^. However, it is worth noting that the molecular mechanisms regulating the protein posttranslational modification of PP2C family protein phosphatases have remained poorly investigated thus far. Exploring the mechanism of PP2C regulation will not only help fully elucidate its function but also provide a reference for other species.

*Ganoderma lucidum*, a traditional Chinese medicinal and food macrofungus, has a variety of pharmacological effects^18–21^. Based on its remarkable physiological activity, ganoderic acid (GA) is an important index to evaluate its quality, but its low content limits the application and development of *G. lucidum*. Furthermore, as a secondary metabolite, the biosynthesis of GA is subject to environmental regulation. Therefore, studying the biosynthetic mechanism of GA is valuable in analyzing the environmental factors that govern the biosynthesis of secondary metabolites. At present, based on developments in genomics and proteomics in recent years and the progress of genetic research methods, there are an increasing number of reports on the mechanism of GA biosynthesis. Previous reports have shown that signaling molecules (reactive oxygen, nitric oxide, calcium ions) and environmental factors (high temperature, nutrients, chemicals) in the GA biosynthesis play an important role in GA biosynthesis^22–28^, indicating that GA biosynthesis is driven by many factors coregulated. Importantly, research on these mechanisms is far from sufficient to reveal the regulatory network of the GA biosynthesis mechanism. Research on the function of epigenetic modification in *G. lucidum* is very limited. Dissecting epigenetic modifications will enrich the regulatory network of the GA biosynthetic mechanism.

In this study, we found that *Gl*PRMT5 negatively regulates the biosynthesis of the secondary metabolite GA in *G. lucidum*. In contrast, *Gl*PP2C1 positively regulates the biosynthesis of secondary metabolites in *G. lucidum*. The activity of the protein phosphatase *Gl*PP2C1 is regulated by arginine methyltransferase *Gl*PRMT5-mediated symmetric dimethylation at R99, a critical residue for its enzymatic activity. In conclusion, on the one hand, we elucidated the target of *Gl*PRMT5 symmetric methylation and its function of modifying proteins by symmetric methylation in large medicinal fungi. On the other hand, we demonstrated that *Gl*PP2C1 is involved in *Gl*PRMT5-mediated GA biosynthesis, providing a mechanism by which PRMT5 regulates secondary metabolites.

## Results

### *GlPRMT5* silencing increases GA content in *G. lucidum*

**Fig. 1:**
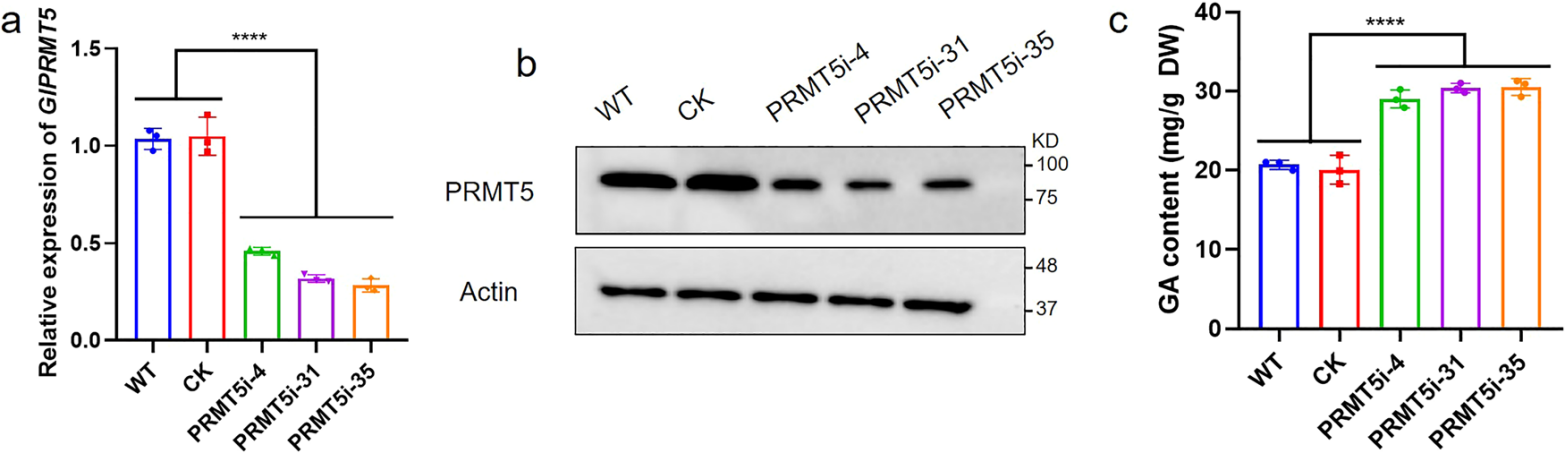
Determination of GA content in the WT, CK and PRMT5-silenced strains. **a** qRT–PCR analysis of the expression of *GlPRMT5* in the tested strains. **b** The PRMT5 protein content in the WT, CK and PRMT5-silenced strains was detected by Western blot analysis. **c** The GA content in the tested strains. The data are presented as the means ± SDs based on three independent experiments (****P < 0.0001 by one-way ANOVA).

Previous studies have reported that PRMT5 is involved in regulating the biosynthesis of secondary metabolites^3^. Of note, the specific mechanism underlying this regulation remains unclear. To explore the role of *Gl*PRMT5 in the biosynthesis of GA, one of the most important secondary metabolites in *G. lucidum*, PRMT5-silenced strains were constructed by cloning PRMT5-silenced sequences from cDNA into RNA interference (RNAi) constructs using cassette plasmids as previously described^29^. Previous studies used real-time reverse transcription PCR (qRT-PCR) to screen out two PRMT5-silenced strains (PRMT5i-31 and PRMT5i-35) with silencing efficiencies of 68% and 72% respectively^30^. Here, we screened a PRMT5-silenced strain (PRMT5i-4) with a silencing efficiency of 54% by qRT-PCR (Fig. 1a). The expression of PRMT5 protein in the WT, CK (empty vector control), and PRMT5-silenced strains was further detected by Western blot analysis (Fig. 1b). The results showed that the PRMT5-silenced strains effectively reduced PRMT5 protein levels compared with the WT and CK strains. These results confirmed the effectiveness of the PRMT5-silenced strains. In addition, the GA content was determined in the WT, CK and PRMT5-silenced strains. We found that compared with the WT and CK strains, the GA content of the PRMT5i-4, PRMT5i-31 and PRMT5i-35 strains increased by 1.4-fold, 1.47-fold and 1.48-fold, respectively (Fig. 1c). The results showed that *GlPRMT5* silencing promoted GA accumulation in *G. lucidum*.

### *Gl*PRMT5 interacts with *Gl*PP2C1 in vivo and in vitro

**Fig. 2:**
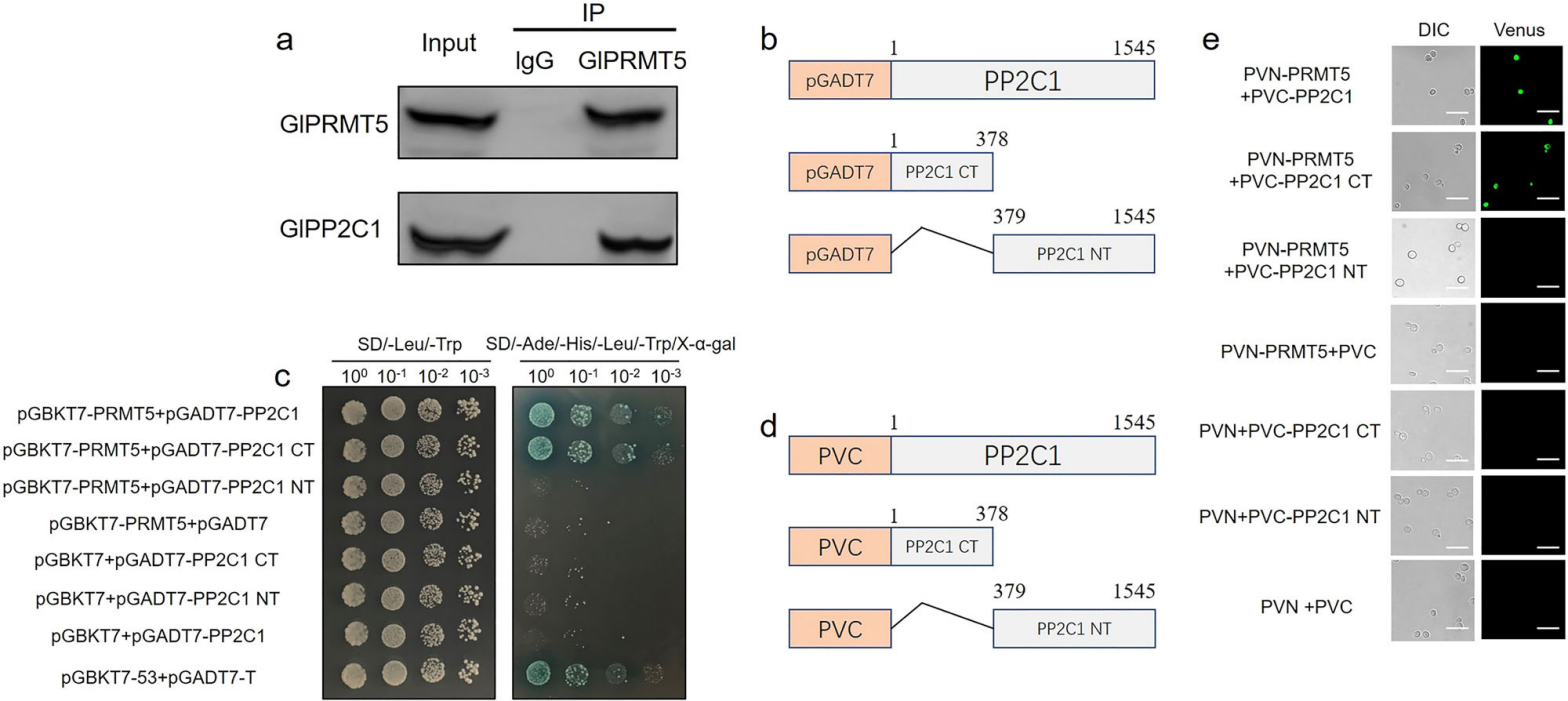
*Gl*PRMT5 interacts with *Gl*PP2C1. **a** Co-IP detection of the interaction between endogenous *Gl*PRMT5 and *Gl*PP2C1. Immunoprecipitation of mycelial lysates with control rabbit IgG or anti-PRMT5 antibody. The IP products were detected by Western blotting with an anti-PRMT5 antibody to assess the accuracy of the experiment. Additionally, the IP product was probed with an anti-*Gl*PP2C1 antibody to specifically detect the interaction between *Gl*PP2C1 and endogenous *Gl*PRMT5. Whole cell extracts (Input) show the results of Western blot with anti-PRMT5 antibody and anti-*Gl*PP2C1 antibody as controls. **b** Schematic representation of the N-terminal (NT) and C-terminal (CT) structures of *Gl*PP2C1 used for Y2H analysis. **c** Y2H assay detection of the interaction between *Gl*PRMT5 and *Gl*PP2C1. pGBKT7-PRMT5, pGADT7-PP2C1, pGADT7-PP2C1 NT, and pGADT7-PP2C1 CT were cotransformed into the Y2H strain. SD-Leu-Trp medium was used for testing successful mating, and SD-Ade-His-Leu-Trp/X-α-gal medium was used for testing interactions. The combination of pGBKT7-53 and pGADT7-T was used as the positive control. **d** Schematic representation of the NT and CT structures of *Gl*PP2C1 used for BiFC analysis. **e** BiFC verified the interaction between *Gl*PRMT5 and *Gl*PP2C1. PVN-PRMT5 containing the GFP tag, PVC-PP2C1, PVC-PP2C1 CT, and PVC-PP2C1 NT were cotransformed into the SFY2620 yeast strain (scale bar = 100 μm). DIC, yeast cell morphology under the normal white field of view; Venus, yeast cell morphology under green fluorescence.

Previous studies have reported that PRMT5 regulates physiological functions by directly binding to nonhistone proteins^7,8^. To study the molecular mechanism by which *Gl*PRMT5 regulates GA biosynthesis, immunoprecipitated proteins were analyzed by mass spectrometry using an anti-PRMT5 antibody. A total of 98 interacting proteins were found by mass spectrometry analysis, among which *Gl*22901 (*Gl*PP2C1 protein phosphatase) had the highest score (Score: the protein matching degree, the higher the score, the higher the reliability, Supplementary Table 1). To investigate the interaction between endogenous *Gl*PRMT5 and *Gl*PP2C1, immunoprecipitation was conducted. After immunoprecipitation with rabbit anti-PRMT5 or normal rabbit IgG beads, the IP product was treated with anti-PRMT5 antibody and subjected to Western blot analysis to assess the accuracy of the experiment. Furthermore, the IP product was also treated with anti-*Gl*PP2C1 antibody to examine the interaction with endogenous *Gl*PRMT5. The results showed that endogenous *Gl*PRMT5 forms a physical complex with endogenous *Gl*PP2C1 in *G. lucidum* (Fig. 2a). Previous studies reported that the cDNA length of *Gl*PP2C1 is 1545 bp, consisting of a nonconserved C-terminal (CT, 1-378 bp) domain and a conserved N-terminal (NT, 379-1545 bp) PP2C_SIG domain^31^. To identify the interaction between *Gl*PRMT5 and *Gl*PP2C1 and identify the specific domain of interaction, the *GlPP2C1* gene was truncated into CT and NT according to the domain (Fig. 2b), and a yeast two-hybrid assay (Y2H) was performed. As shown in Fig. 2c, the yeast strains cotransformed with pGBKT7-PRMT5 and full-length pGADT7-PP2C1 or pGADT7-PP2C1 CT grew well on selective media in the presence of the X-α-D-galactoside (X-α-gal) indicator, which is blue on the plate. The truncated form of pGADT7-PP2C1 NT did not grow on plates containing X-α-gal. This indicated that *Gl*PRMT5 interacts with the full length of *Gl*PP2C1 in yeast and interacts with the CT of PP2C1, rather than the NT of PP2C1. In addition, we further determined the interaction between the *Gl*PRMT5 and *Gl*PP2C1 through a bimolecular fluorescence complementation (BiFC) assay. According to the domain structure, the *GlPP2C1* gene was truncated (Fig. 2d). Yeast strain SFY2620 cotransformed with PVN-PRMT5 and full-length PVC-PP2C1 and PVC-PP2C1 CT fusion vectors exhibited strong green fluorescent protein (GFP) fluorescence signals (Fig. 2e). Notably, the truncated form of PVC-PP2C1 NT showed no signal. Taken together, these results suggested that *Gl*PRMT5 interacts with *Gl*PP2C1 in vivo and in vitro.

### Three-dimensional structure and molecular docking of the *Gl*PRMT5 and *Gl*PP2C1 proteins

**Fig. 3:**
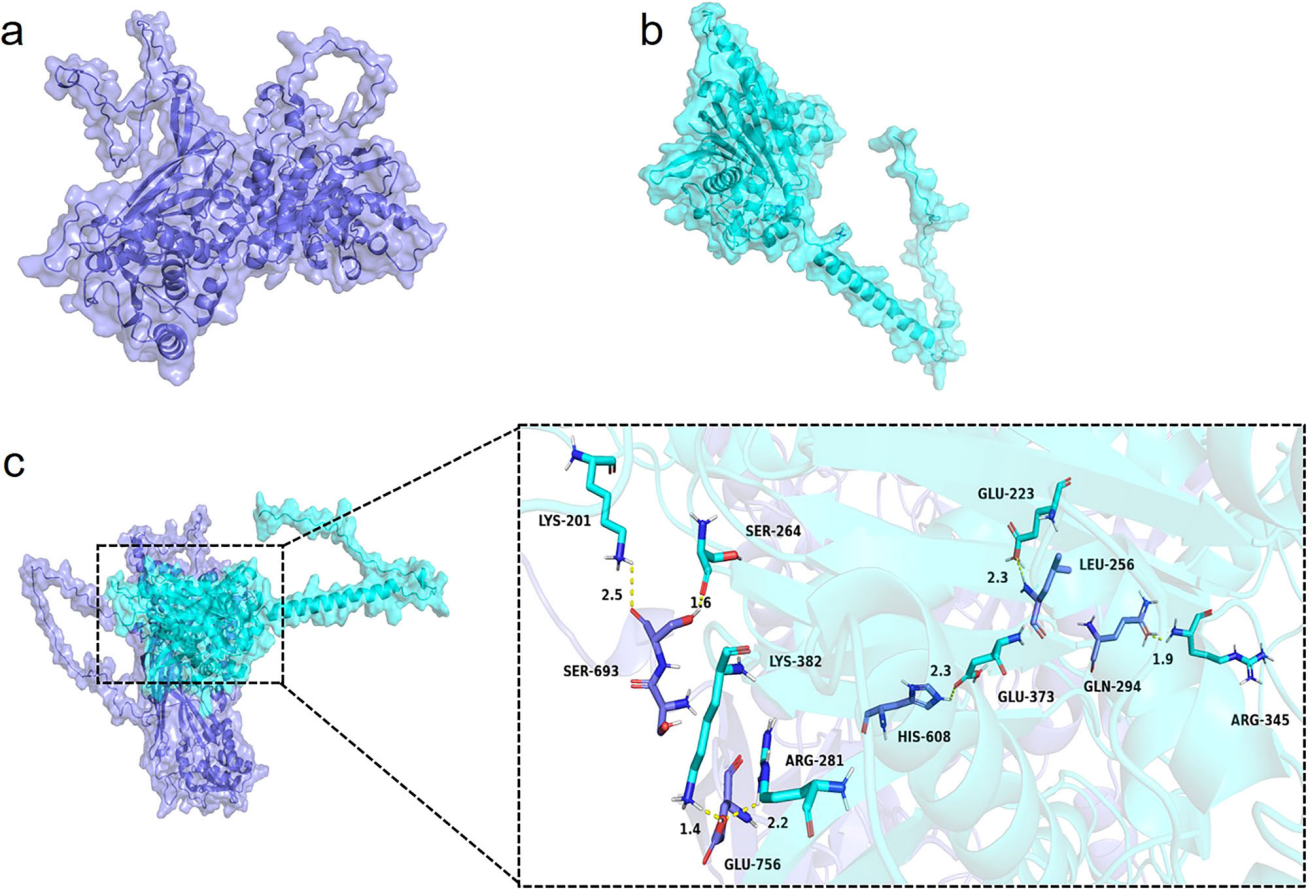
Three-dimensional structure and molecular docking of *Gl*PRMT5 and *Gl*PP2C1 proteins. **a** The protein three-dimensional structure of *Gl*PRMT5 by SWISS-MODEL. **b** The protein three-dimensional structure of *Gl*PP2C1 by SWISS-MODEL. **c** Molecular docking of the *Gl*PRMT5 and *Gl*PP2C1 proteins by AutoDockTools-1.5.7 and PyMol. In PyMol, *Gl*PRMT5 is represented as a dark blue cartoon model, *Gl*PP2C1 is shown as a cyan cartoon model, and their binding sites are shown as stick structures of corresponding colors. When focusing on a binding region, the binding site is displayed with a representation of the protein to which it belongs.

To observe the three-dimensional structure of *Gl*PRMT5 and *Gl*PP2C1 more intuitively, SWISS-MODEL was used. The protein sequence of *Gl*PRMT5 was uploaded to SWISS-MODEL, and the protein with the highest sequence identity (PDB ID: A0A5C3P5V3.1.A, GMQE: 0.85) was selected as a template (Fig. 3a). Similarly, the protein sequence of *Gl*PP2C1 was uploaded to SWISS-MODEL, and the protein with the highest sequence identity (PDB ID: A0A4Q9NLU0, GMQE: 0.81) was selected as a template (Fig. 3b). Molecular docking is one of the most important methods of molecular simulation, helping explore the binding mode and interaction between proteins. To explore the protein interaction between *Gl*PRMT5 and *Gl*PP2C1, molecular docking of *Gl*PRMT5 and *Gl*PP2C1 was performed (Fig. 3c). Using *Gl*PRMT5 as the receptor and *Gl*PP2C1 as the ligand, all functional residues were found by protein‒protein interaction analysis in PyMol. In the hydrogen bond interaction, there are multiple groups of residues used to form hydrogen bonds between *Gl*PRMT5 and *Gl*PP2C1, such as the hydrogen bond formed by HIS608 of *Gl*PRMT5 and GLU373 of *Gl*PP2C1. Under the effect of these interaction forces, *Gl*PRMT5-*Gl*PP2C1 scored −711 and performed better. These results further suggested the interaction of *Gl*PRMT5 and *Gl*PP2C1.

### *Gl*PRMT5 negatively regulates *Gl*PP2C1 protein activity

**Fig. 4:**
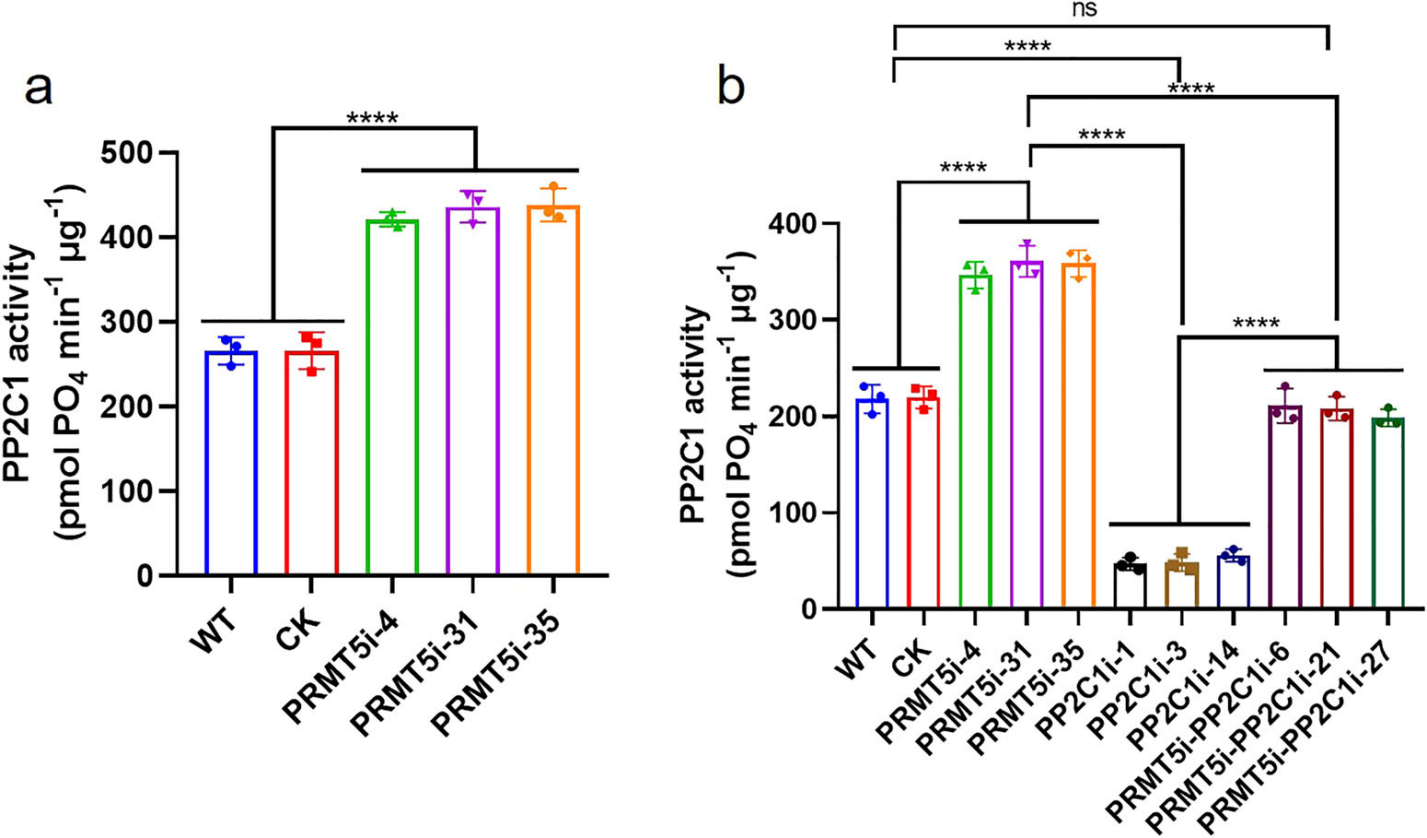
Detection of *Gl*PP2C1 enzyme activity in different strains. **a** Determination of *Gl*PP2C1 enzyme activity in the WT, CK and PRMT5-silenced strains. **b** Determination of *Gl*PP2C1 enzyme activity in the WT, CK, PP2C1-silenced, PRMT5-silenced and PRMT5-PP2C1 cosilenced strains. The data are presented as the means ± SDs based on three independent experiments (ns, not significant; ****P < 0.0001 by one-way ANOVA).

Numerous studies have shown that protein‒protein interactions can plainly affect the activity of target proteins^32,33^. To observe the changes in *Gl*PP2C1 protein activity, *Gl*PP2C1 protein phosphatase activity was detected in the WT, CK and PRMT5i-silenced strains. The data showed that *Gl*PP2C1 activity was evidently increased in the PRMT5-silenced strains, and a 62% increase in *Gl*PP2C1 activity was found in the PRMT5-silenced strains compared with the WT and CK strains (Fig. 4a). This result suggested that silencing *GlPRMT5* increases *Gl*PP2C1 activity.

In addition, PP2C1-silenced strains and PRMT5-PP2C1 cosilenced (PRMT5i-PP2C1i) strains were constructed using the silencing vector of the dual-promoter system previously described by our laboratory^29^. Previous studies used qRT-PCR to screen out two PP2C1-silenced strains (PP2C1i-1 and PP2C1i-3) with silencing efficiencies of 65% and 70%, respectively^31^. Here, we screened a PP2C1-silenced strain (PP2C1i-14) with a silencing efficiency of 62% by qRT-PCR (Supplementary Fig. 1a). The expression of PP2C1 protein in the WT, CK and PP2C1-silenced strains was further detected by Western blot analysis (Supplementary Fig. 1b). The results showed that the PP2C1-silenced strains effectively reduced PP2C1 protein levels compared with the WT and CK strains. Moreover, the structure of the PRMT5i-PP2C1i vector constructed for silencing the expression of *PRMT5-PP2C1* is shown in Supplementary Fig. 1c. Three strains (PRMT5i-PP2C1i-6, PRMT5i-PP2C1i-21 and PRMT5i-PP2C1i-27) were screened by qRT‒PCR among the 38 gene silencing candidate strains (Supplementary Fig. 1d). The expression of PRMT5 and PP2C1 protein in the WT, CK and PRMT5-PP2C1 cosilenced strains was further detected by Western blot analysis (Supplementary Fig. 1e). The results showed that the PRMT5-PP2C1 cosilenced strains obviously reduced PRMT5 and PP2C1 protein levels compared with the WT and CK strains. Taken together, these results confirmed the effectiveness of the PP2C1-silenced strains and the PRMT5-PP2C1 cosilenced strains.

To further observe the changes in *Gl*PP2C1 activity, *Gl*PP2C1 protein phosphatase activity was determined in the WT, CK, PRMT5-silenced, PP2C1-silenced and PRMT5-PP2C1 cosilenced strains (Fig. 4b). The results showed that *Gl*PP2C1 activity in the PRMT5-silenced strains was obviously higher (by 62%) than that in the WT and CK strains, while *Gl*PP2C1 activity in the PP2C1-silenced strains was evidently lower (by 77%) than that in the WT and CK strains. However, *Gl*PP2C1 activity in the PRMT5-PP2C1 cosilenced strains was between that of the PRMT5-silenced strains and PP2C1-silenced strains, and there was no difference between the WT and CK strains. The above results indicate that *Gl*PRMT5 negatively regulates *Gl*PP2C1 protein activity.

### *Gl*PRMT5 symmetrically dimethylates *Gl*PP2C1

**Fig. 5:**
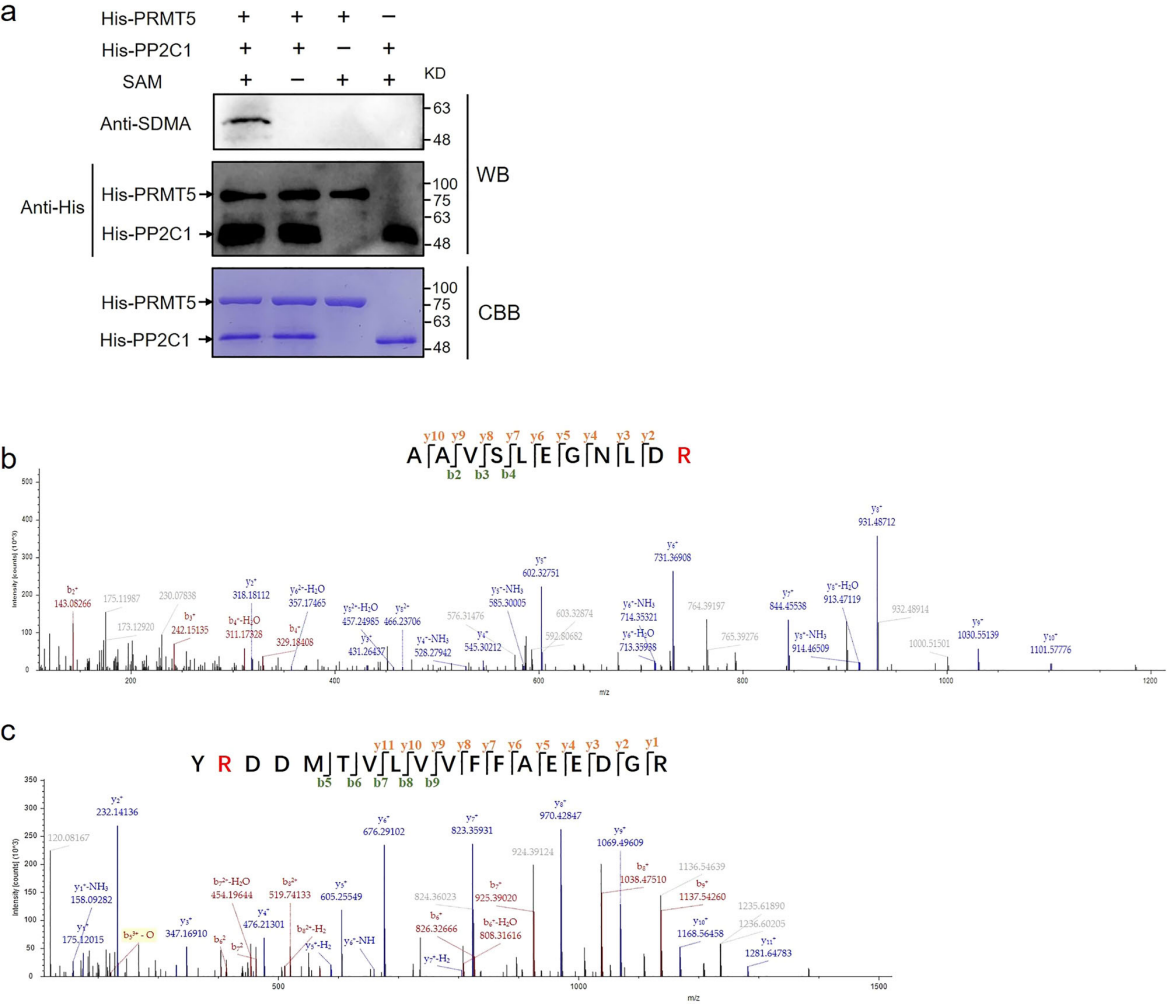
In vitro methylation assays. **a** Purified His-PRMT5 fusion protein was incubated with His-PP2C1 in the presence or absence of SAM. Symmetric dimethylation of His-PP2C1 was detected via Western blot by anti‐SDMA antibody and anti-His, and total amounts of proteins were visualized by Coomassie Blue staining. **b** Mass spectrum of *Gl*PP2C1 Arg 99, AAVSLEGNLDR. **c** Mass spectrum of *Gl*PP2C1 Arg 493, YRDDMTVLVVFFAEEDGR.

This is the most fundamental mechanism by which PRMT5 participates in the signal transduction pathway through symmetric dimethylation of arginine residues in target proteins. To verify the molecular mechanism by which *Gl*PRMT5 regulates *Gl*PP2C1 activity, the pColdI-PRMT5 recombinant protein and pColdI-PP2C1 recombinant protein were expressed and purified by a prokaryotic expression system, and the symmetrical dimethylation of arginine (sDMA) modification status of *Gl*PP2C1 was further analyzed. In the presence of S-adenosyl-methionine (SAM, a methyl donor), *Gl*PRMT5 can symmetrically dimethylate *Gl*PP2C1. In the absence of SAM, when *Gl*PRMT5 was coincubated with *Gl*PP2C1, no signal was detected with the sDMA antibody. Furthermore, no signal was detected when SAM was incubated with *Gl*PRMT5 or *Gl*PP2C1 single protein (Fig. 5a). These results suggested that *Gl*PRMT5 can symmetrically dimethylate *Gl*PP2C1. To further explore potential sites of *Gl*PRMT5-mediated symmetric dimethylation in the His-PP2C1 recombinant protein, the samples after coincubation of *Gl*PRMT5 and *Gl*PP2C1 were analyzed by LC‒MS/MS. The data results showed a series of high-mass-accuracy y ions and b ions identified symmetric dimethylation on arginine 99 (Arg99/R99) and arginine 493 (Arg493/R493) of *Gl*PP2C1 (Fig. 5b, c). The above results indicated that the R99 and R493 sites of the *Gl*PP2C1 protein can be specifically symmetrically dimethylated via *Gl*PRMT5.

### *Gl*PRMT5 catalyzes arginine symmetric dimethylation of *Gl*PP2C1 at the Arg99 and Arg493 residues

**Fig. 6:**
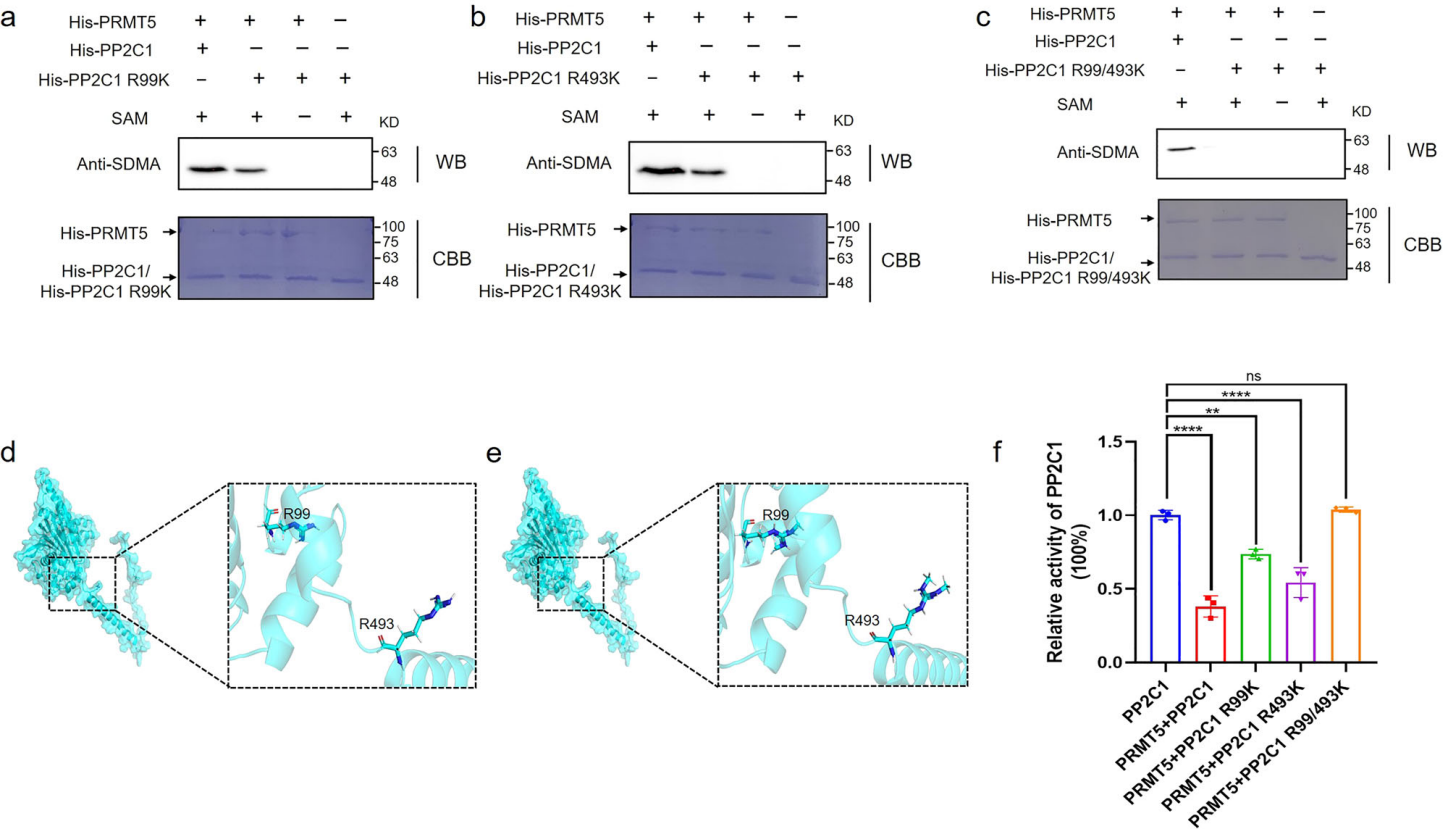
Symmetrical dimethylation modification of *Gl*PP2C1 impairs its enzymatic activity. Purified His-PRMT5 fusion protein was incubated with His-PP2C1, His-PP2C1 R99K (**a**), His-PP2C1 R493K (**b**) or His-PP2C1 R99/493K (**c**) in the presence or absence of SAM. Symmetric dimethylation of His-PP2C1 was detected via Western blot by anti‐SDMA antibody and total amounts of proteins were visualized by Coomassie Blue staining. **d** Unmodified local structure of *Gl*PP2C1 residues R99 and R493 by Schrödinger analysis. **e** Structure of *Gl*PP2C1 R99 and R493 residues modified by symmetric dimethylation via Schrödinger. Gray sticks represent H, cyan sticks represent N, dark blue sticks represent C, and red sticks represent O. **f** Different *Gl*PP2C1 proteins were determined using the Serine/Threonine Phosphatase Assay System as directed by the manufacturer. After the reaction was complete, the absorbance was measured on a microplate reader at a wavelength of 630 nm. The data are presented as the means ± SD based on three independent experiments (ns not significant; **P < 0.01; ****P < 0.0001 by one-way ANOVA).

Since R99 and R493 are the targets of symmetric dimethylation of the *Gl*PP2C1 protein, the symmetric dimethylation modification of these arginine residues may interfere with the *Gl*PP2C1 activity regulated by *Gl*PRMT5. A site-directed mutagenesis kit was used to mutate arginine modified by symmetrical dimethylation into a lysine (R99K and R493K), which cannot be symmetrically dimethylated via *Gl*PRMT5. In addition, a double mutant form (R99/493K) of the recombinant protein was also constructed. The in vitro methylation test was performed on these different forms of recombinant proteins again, and the results showed that the sDMA levels of the single mutant recombinant proteins of His-PP2C1 R99K and His-PP2C1 R493K were evidently lower than those of the unmutated protein His-PP2C1. No signal was detected for the R99/493K double mutant recombinant protein (Fig. 6a–c). In addition, to highlight changes in the structure of *Gl*PP2C1 modified by symmetric dimethylation, Schrödinger analysis was performed. Compared with unmodified *Gl*PP2C1 (Fig. 6d), the R99 and R493 residues of symmetrically dimethylated *Gl*PP2C1 had two symmetrical methyl groups added (Fig. 6e). Changes in protein structure are accompanied by changes in activity. *Gl*PP2C1 activity in different mutant recombinant protein bodies was further determined by the Serine/Threonine Phosphatase Assay System, and the standard curve of absorbance of free phosphate concentration at 630 nm is shown in Supplementary Fig. 2a. In the presence of equal amounts of serine/threonine phosphopeptide substrates, the absorbance gradually increased with increasing amounts of His-PP2C1 protein (Supplementary Fig. 2b), demonstrating that *Gl*PP2C1 possesses phosphatase activity in vitro. In addition, the results showed that the addition of *Gl*PRMT5 plainly reduced *Gl*PP2C1 activity (by 62%) compared to the untreated *Gl*PP2C1 control (Fig. 6f). However, there was a marked increase in *Gl*PP2C1 activity in the single mutant proteins *Gl*PP2C1 R99K body (1.9-fold) and *Gl*PP2C1 R493K body (1.4-fold). Interestingly, *Gl*PP2C1 activity in the double mutant protein body *Gl*PP2C1 R99/493K was consistent with the activity in the control group where only a single *Gl*PP2C1 protein was present. Therefore, the symmetrical dimethylation modification of *Gl*PP2C1 obviously inhibits PP2C activity, and R99 of *Gl*PP2C1 is the key site for its activity. In summary, the above results indicated that *Gl*PRMT5-mediated symmetric dimethylation reduces *Gl*PP2C1 activity, and the modification of the R99 site is the key to affecting *Gl*PP2C1 activity.

### Symmetrical dimethylation of *Gl*PP2C1 does not affect the interaction with *Gl*PRMT5

**Fig. 7:**
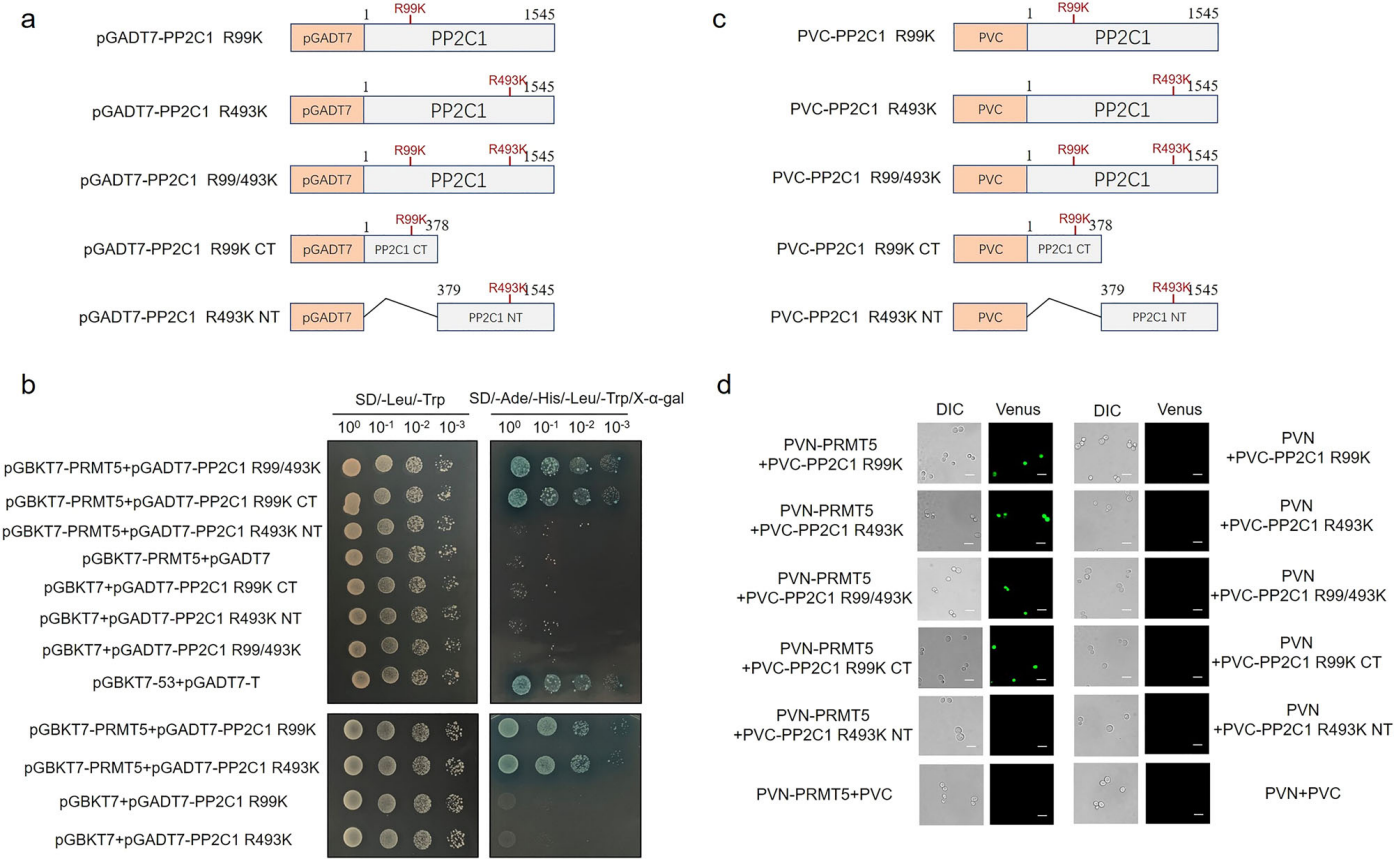
Symmetrical dimethylation of *Gl*PP2C1 does not affect the interaction with *Gl*PRMT5. **a** Schematic representation of NT and CT structures of *Gl*PP2C1 single mutant (R99K, R493) and double mutant (R99/493K) for Y2H analysis. **b** pGBKT7-PRMT5, pGADT7-PP2C1 R99K, pGADT7-PP2C1 R493K, pGADT7-PP2C1 R99/493K, pGADT7-PP2C1 R99K CT, and pGADT7-PP2C1 R493K NT were cotransformed into the Y2H strain. SD-Leu-Trp medium was used for testing successful mating, and SD-Ade-His-Leu-Trp/X-α-gal medium was used for testing interactions. The combination of pGBKT7-53 and pGADT7-T was used as the positive control. **c** Schematic representation of NT and CT structures of *Gl*PP2C1 single mutant (R99K, R493) and double mutant (R99/493K) for BiFC analysis. **d** PVN-PRMT5 containing the GFP tag, PVC-PP2C1 R99K, PVC-PP2C1 R493K, PVC-PP2C1 R99/493K, PVC-PP2C1 R99K CT, and PVC-PP2C1 R493K NT were cotransformed into the SFY2620 yeast strain (scale bar = 100 μm). DIC, yeast cell morphology under the normal white field of view; Venus, yeast cell morphology under green fluorescence.

Symmetrical dimethylation modification of *Gl*PP2C1 reduces its activity via *Gl*PRMT5. To test whether this modification affects the binding between *Gl*PRMT5 and *Gl*PP2C1, *Gl*PP2C1 single mutants (R99K, R493K) and double mutants (R99/493K) were inserted into pGBKT7 and PVC1 (Fig. 7a, c), and further Y2H and BiFC were performed. As shown in Fig. 7b, two single mutant full-length (pGADT7-PP2C1 R99K, pGADT7-PP2C1 R493K) or double mutant full-length (pGADT7-PP2C1 R99/493K) cotransformed with pGBKT7-PRMT5 grew well on the selective medium and appeared blue on the plate containing X-α-gal. In addition, the truncated form of pGADT7-PP2C1 R99K CT, a cotransformed in yeast strain with pGBKT7-PRMT5, grew well on selective medium and appeared blue on plates containing X-α-gal, while the truncated form of pGADT7-PP2C1 R493K NT did not grow on plates containing X-α-gal. In addition, BiFC was conducted to further determine the interaction between *Gl*PRMT5 and *Gl*PP2C1 modified by symmetric dimethylation. Yeast strain SFY2620 cotransformed with PVN-PRMT5 and two single mutant full-length (PVC-PP2C1 R99K, PVC-PP2C1 R493K) or double mutant full-length (PVC-PP2C1 R99/493K) fusion vectors exhibited strong GFP fluorescence signals (Fig. 7d). In addition, the truncated form of PVC-PP2C1 R99K CT, cotransformed in yeast strain SFY2620 with PVN-PRMT5, exhibited strong GFP fluorescence signals. However, the truncated form of PVC-PP2C1 R493K NT showed no signal. These results indicated that *Gl*PP2C1 modified by symmetrical dimethylation did not affect the interaction with *Gl*PRMT5.

### Silencing *GlPP2C1* results in reduced GA content

**Fig. 8:**
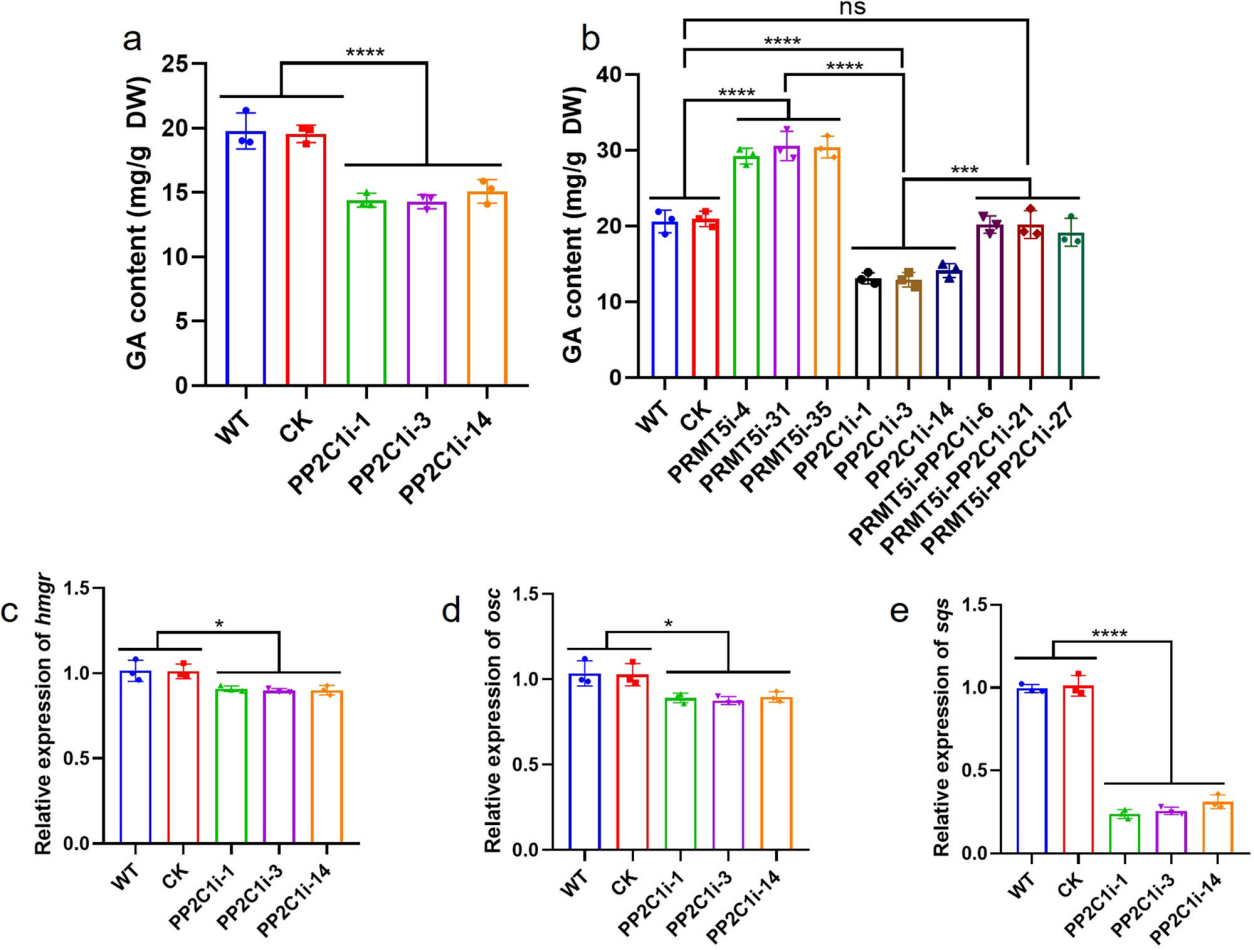
Determination of GA content in different strains. **a** Determination of GA content in the WT, CK and PP2C1-silenced strains. **b** Determination of GA content in the WT, CK, PP2C1-silenced, PRMT5-silenced and PRMT5-PP2C1 cosilenced strains. **c** Relative expression levels of *hmgr* in the WT, CK and PP2C1-silenced strains. **d** Relative expression levels of *osc* in the WT, CK and PP2C1-silenced strains. **e** Relative expression levels of *sqs* in the WT, CK and PP2C1-silenced strains. The data are presented as the means ± SDs based on three independent experiments (ns not significant; *P < 0.1; ***P < 0.001; ****P < 0.0001 by one-way ANOVA).

GA is the most important secondary metabolite in *G. lucidum*. To explore whether *Gl*PP2C1 is involved in regulating the GA biosynthesis, the GA content was determined in the WT, CK and PP2C1-silenced strains. We found that the GA content in the PP2C1-silenced strains was plainly decreased (by approximately 33%), compared with that in the WT and CK strains (Fig. 8a). This result indicated that *Gl*PP2C1 played a positive regulatory role in the GA biosynthetic pathway. To elucidate the role of *Gl*PP2C1 in *Gl*PRMT5-regulated GA biosynthesis, the GA content was examined in the WT, CK, PP2C1-silenced, PRMT5-silenced and PRMT5-PP2C1 cosilenced strains (Fig. 8b). The results showed that the GA content in the PRMT5-PP2C1 cosilenced strains was between the PRMT5-silenced strains and the PP2C1-silenced strains, consistent with the content in the WT and CK strains. Previous studies have suggested that GA is synthesized via the mevalonate/isoprenoid pathway, wherein hydroxy-3-methylglutaryl-CoA reductase (*hmgr*), 2,3-oxidosqualene-lanosterol cyclase (*osc*) and squalene synthase (*sqs*) play crucial roles in this pathway^34–38^. The key genes expression levels of GA biosynthesis (*hmgr*, *osc* and *sqs*) were detected in the WT, CK and PP2C1-silenced strains. The results revealed that the expression of *hmgr* and *osc* in the PP2C1-silenced strains were not obviously different from those in the WT and CK strains (Fig. 8c, d), while the expression of *sqs* exhibited a decrease of 70% in the PP2C1-silenced strains in comparison to the WT and CK strains (Fig. 8e). The above results suggested that silencing *GlPP2C1* results in reduced GA content.

## Discussion

PRMT5, a type II arginine methyltransferase, can perform symmetric dimethylation on the arginine sites of histones and nonhistones^39^. Compared with the research on histones, there are very few studies on the regulation of PRMT5 on nonhistones. With the development of science, technology and instruments, a series of target nonhistones of PRMT5 have been gradually identified. For example, MCM7 (minichromosome maintenance-7) was identified as a direct PRMT5 binding partner via Co-IP and MS analysis^40^. MTHFD1 (methylenetetrahydrofolate dehydrogenase, cyclohydrolase and formyltetrahydrofolate synthetase 1) can specifically bind PRMT5^41^. Discovering target proteins of PRMT5 is the key to exploring its function. Here, we report for the first time the target protein *Gl*PP2C1 of *Gl*PRMT5 by MS analysis in *G. lucidum*. In addition, PRMT5 was shown to affect the activity, localization, and function of target proteases by binding to nonhistone proteins and undergoing symmetrical dimethylation modification^42^. For example, PRMT5 promotes AKT activation by symmetrically dimethylating AKT protein kinase to control tumorigenesis^33^. *At*PRMT5-mediated *At*LCD (l-cysteine desulfhydrase) methylation increases its enzymatic activity, thereby enhancing endogenous H_2_S signaling and ultimately improving plant tolerance to Cd^2+^ stress in *Arabidopsis*^43^. In this study, it was found that *Gl*PRMT5-mediated symmetric dimethylation of *Gl*PP2C1 reduced *Gl*PP2C1 enzymatic activity. The interaction of *Gl*PRMT5 and *Gl*PP2C1 may regulate the biosynthesis of secondary metabolites.

A large number of existing studies have reported that type 2C protein phosphatases are involved in signal transduction and biological processes. For example, overexpression of *PtrHAB2* (a type 2C protein phosphatase) leads to an increased plant growth rate, increased trunk height, and altered leaf morphogenesis in *Populus trichocarpa*^44^. Genome-wide expression analysis in rice revealed that transcripts of several PP2C genes were obviously altered during critical stages of reproductive development^45^. Biosynthesis of the secondary metabolite deoxynivalenol is noticeably reduced in 11 phosphatase deletion mutants of *Fusarium graminearum*^46^. *Aspergillus fumigatus* protein phosphatase PpzA is involved in affecting secondary metabolites^47^. Type 2C protein phosphatases PTC1 and PTC2 in *Aspergillus flavus* positively regulate the content of secondary metabolite aflatoxin^15^. In our study, silencing *GlPP2C1* resulted in a remarkable decrease in GA content, suggesting that *Gl*PP2C1 positively regulates GA biosynthesis. This indicates that protein phosphatase generally has the function of regulating fungal secondary metabolism. It was further found that the transcription level of key genes for GA biosynthesis (*sqs*) was notably decreased in the PP2C1-silenced strains. This indicates that *Gl*PP2C1 may affect the biosynthesis of secondary metabolites of *G. lucidum* by regulating the key genes expression level of GA biosynthesis. Until now, PP2C has also been reported to regulate the biosynthesis of secondary metabolites in various ways. For instance, type 2C protein phosphatase regulates the stability of ACS7, the rate-limiting enzyme in ethylene biosynthesis in *Arabidopsis*^48^. Type 2C phosphatases Ptc1 and Ptc2 participate in autophagy and mitochondrial pyruvate metabolism through dephosphorylation of phosphoglycerate kinase 1 (PGK1) in *A. flavus*, thereby regulating aflatoxin synthesis^15^. Type 2C protein phosphatase *Gh*DRP1 in cotton affects ROS scavenging enzymes and the accumulation of proline by regulating the flavonoid biosynthetic pathway^49^. This indicates that *Gl*PP2C1 may also affect the secondary metabolism and biosynthesis of *G. lucidum* through other modes of action. Furthermore, it has been widely reported that type 2C protein phosphatases perform their main function by dephosphorylating substrate protein kinases. For example, Ptc1, a type 2C Ser/Thr phosphatase, inactivates the HOG pathway by dephosphorylating the mitogen-activated protein kinase (MAPK) in *S. cerevisiae*^50^. The PP2C enzyme deactivates MAPKs through dephosphorylation, and thus blocks the downstream regulation of the signaling cascade^51^. Therefore, it is necessary to study the molecular mechanism by which *Gl*PP2C1 regulates GA biosynthesis in *G. lucidum*.

Most studies have screened the downstream proteins of PP2C^14,15^, but there are still few studies on the proteins regulating PP2C. Upon light exposure, SAUR50 binds and inhibits PP2C-D1 activity, inducing general cell expansion and ultimately leading to hook and cotyledon opening and cotyledon enlargement in *Arabidopsis*^52^. In addition, regulatory mechanisms of type 2 A protein phosphatases have been extensively reported^53,54^. SBI1, encodes a leucine carboxylmethyltransferase (LCMT) that methylates PP2A, thereby promoting its association with activated BRI1 in *A. thaliana*^55^. PRMT5 negatively regulates the enzymatic activity of PP2A^56^. Therefore, it is necessary to explore the mechanism regulating type 2C protein phosphatase activity. It has been reported that the activity of type 2C protein phosphatase is regulated through protein post-translational modification. For instance, the H_2_O_2_ produced by aba-induced NADPH oxidase RbohB/E inhibits PP45 activity by oxidizing Cys-350 and Cys-428 residues of PP45^16^. Our study showed that *Gl*PRMT5 mediates symmetrical dimethylation of *Gl*PP2C1 at residues R99 and R493, resulting in decreased *Gl*PP2C1 activity. The study identifies the upstream protein of *Gl*PP2C1, providing more meaningful insights into the molecular mechanism of PP2C in other species.

With recent advances in proteomics technology, especially mass spectrometry, a large number of studies have shown that many proteins are cotargeted by many different types of posttranslational modifications to regulate protein function. For example, PRMT5 symmetrically dimethylates ASK1 (apoptosis signal-regulating kinase 1) at the arginine 89 residue, thereby promoting the interaction between ASK1 and Akt and phosphorylating ASK1 at the serine 83 residue to negatively regulate its activity^57^. PRMT5-mediated symmetric dimethylation of KLF5 (an oncogenic factor) at R57 antagonizes GSK3β-mediated KLF5 phosphorylation and Fbw7-mediated KLF5 ubiquitination to promote basal-like breast cancer (BLBC)^58^. It is worth noting that PRMT5 has been widely reported to have diverse functions and can participate in the same biological process through multiple target proteins. For instance, PRMT5 interacts with and methylates Mxi1, thereby promoting the binding of β-Trcp ligase to Mxi1 and promoting the ubiquitination and degradation of Mxi1 in lung cancer^59^. PRMT5 methylates the oncogenic factor Krüppel-like factor 5 (KLF5) to prevent its degradation, thereby promoting the maintenance and proliferation of lung cancer cells^60^. In our study, we found that *Gl*PRMT5-mediated symmetric dimethylation of *Gl*PP2C1 markedly decreased its activity and GA content. Furthermore, the GA content and *Gl*PP2C1 activity in the PRMT5-PP2C1 cosilenced strains were higher than those in the PP2C1 alone-silenced strains, indicating that in addition to *Gl*PP2C1, *Gl*PRMT5 may have other pathways to regulate GA biosynthesis. This may be the result of the crosstalk formed by methylation and dephosphorylation jointly regulating GA biosynthesis. This will also be the focus of our further research.

Collectively, our research results showed that *Gl*PP2C1, the interaction target protein of *Gl*PRMT5, has been identified. The interaction between *Gl*PRMT5 and *Gl*PP2C1 was verified in vivo and in vitro. Furthermore, on the one hand, our data suggested that *Gl*PRMT5-mediated symmetric dimethylation negatively regulates *Gl*PP2C1 activity. On the other hand, it was demonstrated that *Gl*PP2C1 is involved in *Gl*PRMT5-mediated GA biosynthesis. *Gl*PRMT5-mediated symmetric dimethylation of residues R99 and R493 in *Gl*PP2C1 clearly attenuated *Gl*PP2C1 activity. Further data indicated that the R99 residue in *Gl*PP2C1 is the key to affecting *Gl*PP2C1 activity. In addition, the symmetrical dimethylation modification did not affect the interaction between *Gl*PP2C1 and *Gl*PRMT5. These data establish a framework for the regulation of secondary metabolites via *Gl*PRMT5 in macrofungi. The identification of modified proteins and characteristic sites is conducive to a comprehensive understanding of the molecular mechanism and regulatory functions of *Gl*PRMT5-mediated symmetric dimethylation.

## Methods

### Strains and culture conditions

The wild-type (WT) strain was selected from *G. lucidum* ACCC53264 provided by the Agricultural Culture Collection of China. The PP2C1-silenced strains (PP2C1i-1 and PP2C1i-3) and PRMT5-silenced strains (PRMT5i-31 and PRMT5i-35) were established in our previous study^30,31^. The strains were fermented in CYM broth for 7 days at 28 °C in a shaking incubator at 150 rpm to measure GA content.

### Construction of RNAi strains

*G. lucidum* cDNA was used as a template to amplify the *Gl*PRMT5 gene and *Gl*PP2C1 gene fragments, and PP2C1i-F (GGGGTACACTCGCATTCCCGCTT), PP2C1i-R (CGCCTCTCCGTTCATTGCCCTGTGCTTT), PRMT5i-F (AAAGGCACCAGGCACATGAAACGGGCG) and PRMT5i-R (GACTAGTAGCGTGGGTATGTGGG) were used for joint PCR to construct fungal RNAi vectors^61^. The RNAi silencing vector pAN7-dual-PRMT5i-PP2C1i was electroporated into *G. lucidum*, and three independent strains with the highest silencing efficiency were selected for subsequent experiments^25^. The empty vector control was named CK.

### Gene expression analysis

Using the cDNAs of different transformants screened as templates and the primers listed in Supplementary Table 2, quantitative RT‒qPCR analysis was performed using Eppendorf Mastercycler Ep Realplex 2.2 software. The transcript level was analyzed for gene-specific mRNAs using 18 S rRNA as the housekeeping gene as previously described^62^. The relative gene expression levels were determined using the 2^−ΔΔCT^ method.

### Extraction and detection of GA

GA was extracted and detected as previously described^22^. In brief, dried mycelial powder (200 mg) was ultrasonically extracted in 10 mL of 95% ethanol for 2 h. The mixture was centrifuged at 4000 rpm for 10 min to obtain the supernatant. Next, the supernatant was dried with a rotary evaporator to obtain the crude extract. The crude extract was then resuspended in 0.5 mL methanol and analyzed with ultraperformance liquid chromatography (UPLC).

### Expression and purification of recombinant proteins and antibody preparation

*Gl*PRMT5 and *Gl*PP2C1 sequences from *G. lucidum* were deposited in GenBank (OP360010 and OP251201, respectively). The expression vectors pColdI-PRMT5, pColdI-PP2C1, pColdI-PP2C1 R99K, pColdI-PP2C1 R493K and pColdI-PP2C1 R99/493K were transformed into *E. coli* strain BL21 (DE3), protein expression was induced with 500 µM isopropyl-β-D-thiogalactopyranoside (IPTG), and the strains were grown for 16 h at 16 °C. Purification of recombinant proteins was performed using nickel-nitrilotriacetate (Ni-NTA) agarose columns (Sangon, C600033). In addition, the purified His-PP2C1 recombinant protein (Supplementary Fig. 3) was sent to a professionally qualified antibody preparation company for immunization of rabbits (Chemgen Biotech)^63^.

### Phosphatase activity assay

Protein was extracted from PRMT5-silenced strains or PRMT5-PP2C1 cosilenced strains with phosphatase storage buffer (10 mM Tris, pH 7.5, 1 mM EDTA, 0.02% [w/v] sodium azide). The assay was then performed using the Serine/Threonine Phosphatase Assay System (Promega, Cat# V2460) as directed by the manufacturer. After the reaction was complete, the absorbance was measured on a microplate reader at a wavelength of 630 nm. The purified protein (pColdI-PP2C1, pColdI-PP2C1 R99K, pColdI-PP2C1 R493K and pColdI-PP2C1 R99/493K) was also measured as above.

### In vitro methylation assay

The purified pColdI-PRMT5 in pColdI-PP2C1, pColdI-PP2C1 R99K, pColdI-PP2C1 R493K and pColdI-PP2C1 R99/493K were incubated with 50 μL of reaction buffer (20 mM Tris-HCl (pH 7.5), 150 mM NaCl, 2 mM EDTA, 1 mM PMSF, 1 mM DTT), with or without 16 mmol/L methyl donor S-adenosyl-methionine (SAM, Solarbio, S9990). Then, the cells were incubated at 30 °C for 90 min. The reactions were stopped by the addition of sample loading buffer, followed by immunoblotting with anti-sDMA antibody (anti-dimethyl-arginine, symmetric, SYM11, Millipore, 07-413).

### Mass spectrometry (MS) analysis

After incubating the purified pColdI-PRMT5, pColdI-PP2C1 and SAM in the above 50 μL reaction buffer, they were treated at 30 °C for 90 min and separated on a 12% (wt/vol) SDS‒PAGE gel. The *Gl*PP2C1 protein was cleaved, digested with trypsin in the gel, and analyzed by LC‒MS/MS analysis of polypeptides. Peptide identification was performed using MS/MS spectra of the *Gl*PP2C1 protein.

### Yeast two-hybrid assay

The full-length *Gl*PRMT5 gene was inserted into pGBKT7 to construct the bait vector. We inserted the full-length *Gl*PP2C1 gene and the truncated versions of PP2C1 CT and PP2C1 NT into pGADT7 to construct the prey vector. The bait and prey vectors were cotransformed into the yeast Y2HGold strain and cultured in SD/-Leu/-Trp medium and SD/-Ade-His-Leu-Trp/X-α-Gal medium at 30 °C for 3 to 5 days. Assessment of protein interaction is based on the development of blue colonies on the plate. Y2H interactions between two single mutant full-length proteins (pGADT7-PP2C1 R99K, pGADT7-PP2C1 R493K), double mutant full-length proteins (pGADT7-PP2C1 R99/493 K), the truncated form of pGADT7-PP2C1 R99K CT and pGADT7-PP2C1 R493K NT and pGBKT7-PRMT5 were detected by the above method.

### BiFC assay

Bimolecular fluorescence complementation (BiFC) assays were performed as follows^64^. Briefly, the full-length *Gl*PRMT5 gene was inserted into pVN1, and full-length *Gl*PP2C1 and truncated forms of PP2C1 CT and PP2C1 NT were inserted into pVC1. The recombinant constructs were subsequently cotransformed into yeast strain SFY2620 and incubated in SD/-Leu-Ura medium at 30 °C for 3 days. Yeast strains were evaluated for green fluorescence by confocal microscopy. The BiFC interaction between two single mutant full-length proteins (PVC-PP2C1 R99K, PVC-PP2C1 R493K), double mutant full-length proteins (PVC-PP2C1 R99/493K), the truncated forms of PVC-PP2C1 R99K CT and PVC-PP2C1 R493K NT and PVN-PRMT5 were detected by the above method.

### Coimmunoprecipitation assay

Coimmunoprecipitation was carried out according to a previously reported method in the laboratory^61^. Briefly, *G. lucidum* mycelia powder (0.15 g) was lysed in lysis buffer containing 100 mM NaCl, 20 mM Tris–HCl pH 7.6, 0.1% Triton X-100, 0.5% protease inhibitor cocktail (Sigma), and 1 mM phenylmethanesulfonyl fluoride (Sangon) for 1 h. The supernatants (200 µL) were immunoprecipitated with protein A/G agarose (25 µL, Thermo Scientific, 88802), followed by incubation with control rabbit IgG (Solarbio) or PRMT5 antibody (Abcam, ab109451) at 4 °C overnight. The immunoprecipitated proteins were was washed (5 times) with lysis buffer, and the protein lysate was further eluted with 5× SDS loading buffer. Western blotting was further performed using anti-PRMT5 antibody and anti-*Gl*PP2C1 polyclonal antibody. In addition, using the same method to immunoprecipitate interacting proteins with PRMT5, the proteins were separated on a 12% (wt/vol) SDS‒PAGE gel and stained with Coomassie blue. Bands were excised, followed by digestion and plastid analysis.

### Homology modeling and molecular docking

The protein sequence information of *Gl*PRMT5 and *Gl*PP2C1 was uploaded to the online website SWISS-MODEL to construct the three-dimensional structure of *Gl*PRMT5 and *Gl*PP2C1. The protein with the highest sequence identity (GMQE value close to 1, high reliability) was selected as the template. Using *Gl*PRMT5 as the receptor and *Gl*PP2C1 as the ligand, AutoDockTools-1.5.7 was used for molecular docking^65^. The default hydrogen bond distance is 2.5 Å, but when the two forces are weak, the distance is set to 3.0 Å, and when the two forces are strong, the distance is set to 2.0 Å. Then, the docking server (GRAMM) was used for protein‒protein docking^66,67^. The obtained protein‒protein complexes were also optimized with AutoDockTools-1.5.7 for dehydration and hydrogenation. Finally, PyMol was used to predict protein interactions and generate protein‒protein interaction maps. Structures of the unmodified *Gl*PP2C1 and symmetric dimethylation modified *Gl*PP2C1 R99 and R493 residues were illustrated using Schrödinger.

### Statistics and reproducibility

Statistical analysis was performed using GraphPad Prism 8 on the data presented in this article, which were obtained from at least three independent samples. Error bars indicate the standard deviation (SD) of triplicate means. Differences in means were analyzed by one-way or two-way analysis of variance (ANOVA) for differences between groups using GraphPad Prism. *p < 0.05, **p < 0.01, ***p < 0.001 and ****p < 0.0001 are statistically significant. NS indicates not significant.

### Reporting summary

Further information on research design is available in the Nature Portfolio Reporting Summary linked to this article.

### Supplementary information

Supplementary Information

Description of Additional Supplementary Files

Supplementary Data 1

Reporting Summary

## Data Availability

All data generated or analyzed during this study are included in this published article (and its Supplementary Information files) or are available from the corresponding author on reasonable request. The source data underlying the graphs in the figure are shown in Supplementary Data 1. The mass spectrometry proteomics data have been deposited to the ProteomeXchange Consortium via the PRIDE partner repository with the dataset identifier PXD048799. Uncropped western blots and gel are in Supplementary Figs. 5–9.
