# Supplementary material for: GlPRMT5 inhibits GlPP2C1 via symmetric dimethylation and regulates the biosynthesis of secondary metabolites in Ganoderma lucidum
Source: Commun Biol. 2024 Feb 28;7:241. doi: 10.1038/s42003-024-05942-y (PMC10902306; doi:10.1038/s42003-024-05942-y)
Supplement: Supplementary file 1 — Supplementary Information [file 42003_2024_5942_MOESM1_ESM.pdf]

Supplementary material for

*Gl*PRMT5 inhibits *Gl*PP2C1 via symmetric dimethylation and regulates the biosynthesis of secondary metabolites in *Ganoderma lucidum*

Zi Wang[1], Hao Qiu[1], Yefan Li[1], Mingwen Zhao*[1], Rui Liu*[1]

[1]Key Laboratory of Agricultural Environmental Microbiology, Ministry of Agriculture and Rural Affairs; Microbiology Department, College of Life Sciences, Nanjing Agricultural University, Nanjing 210095, Jiangsu, P.R. China

*Corresponding author: Mingwen Zhao and Rui Liu. These authors contributed equally.

E-mail: ruiliu@njau.edu.cn

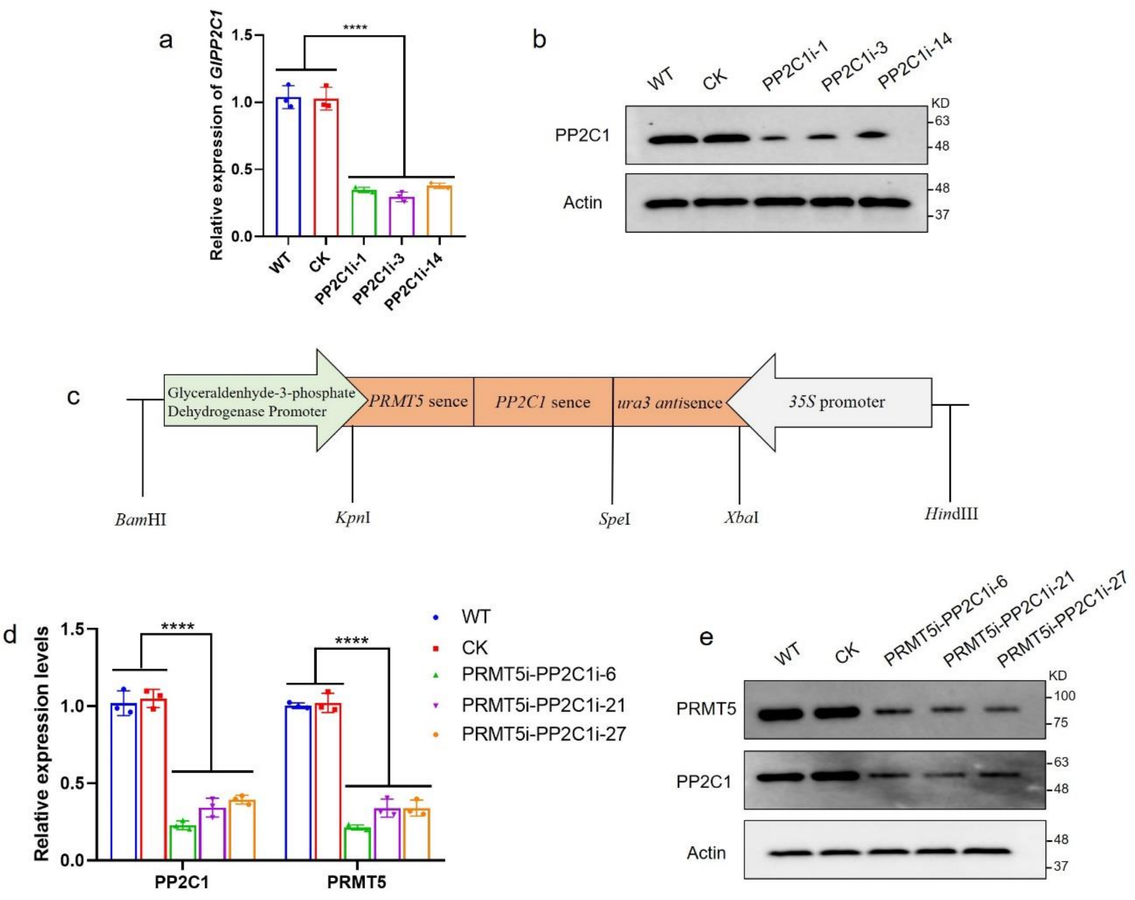

Supplementary Figure 1 Construction of PP2C1-silenced strains and PRMT5-PP2C1 cosilenced strains. (a) qRT–PCR analysis of the expression of *GlPP2C1* in the tested strains. (b) The PP2C1 protein content in the WT, CK and PP2C1-silenced strains was detected by Western blot analysis. (c) Structure of the vector constructed for silencing the expression of *PRMT5-PP2C1*. (d) qRT–PCR analysis of the expression of *Gl*PP2C1 and *Gl*PRMT5 in the tested strains. (e) The PRMT5 and PP2C1 protein content in the WT, CK and PRMT5-PP2C1 cosilenced strains was detected by Western blot analysis. The data are presented as the means ± SD based on three independent experiments (ns, not significant; ****P < 0.0001 by one-way ANOVA).

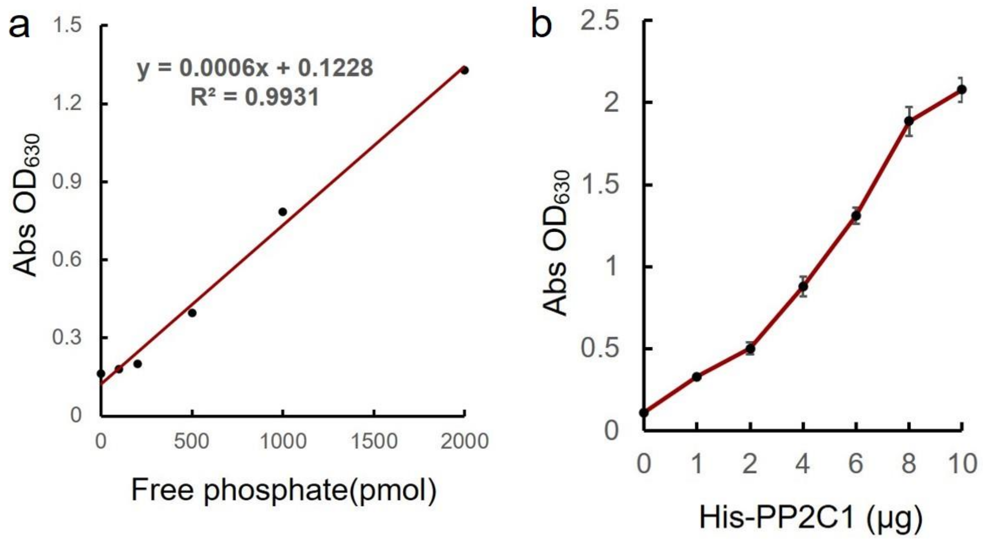

Supplementary Figure 2 *Gl*PP2C1 enzyme activity detection Serine/Threonine Phosphatase Assay System. (a) Standard Curves of Absorbance at 630 nm Versus Concentration of Free Phosphate. (b) *Gl*PP2C1 has phosphatase activity *in vitro*. The fusion protein His-PP2C1 was expressed and purified by prokaryotic expression system for the determination of phosphatase activity. The absorbance at 630 nm indicated its catalytic rate.

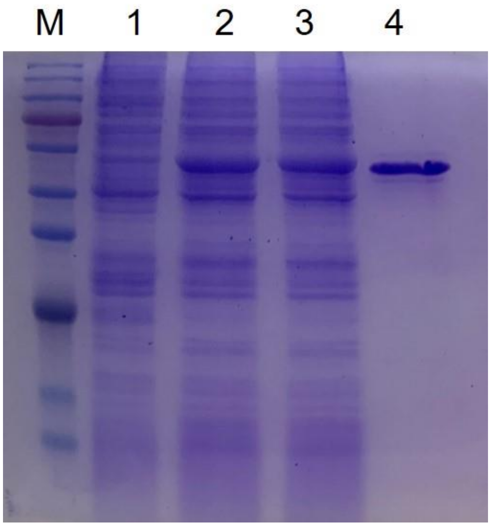

Supplementary Figure 3 SDS-PAGE analysis for *Gl*PP2C1 heterologously expressed in the *E. coli* strain BL21 (DE3). Lane M: protein marker; lane 1: whole cell lysate of recombinant *Gl*PP2C1; lane 2: whole cell lysate of recombinant *Gl*PP2C1 after IPTG treatment; lane 3, supernatant of *Gl*PP2C1 cell lysate; lane 4, purified *Gl*PP2C1.

Source data for Figure 1b

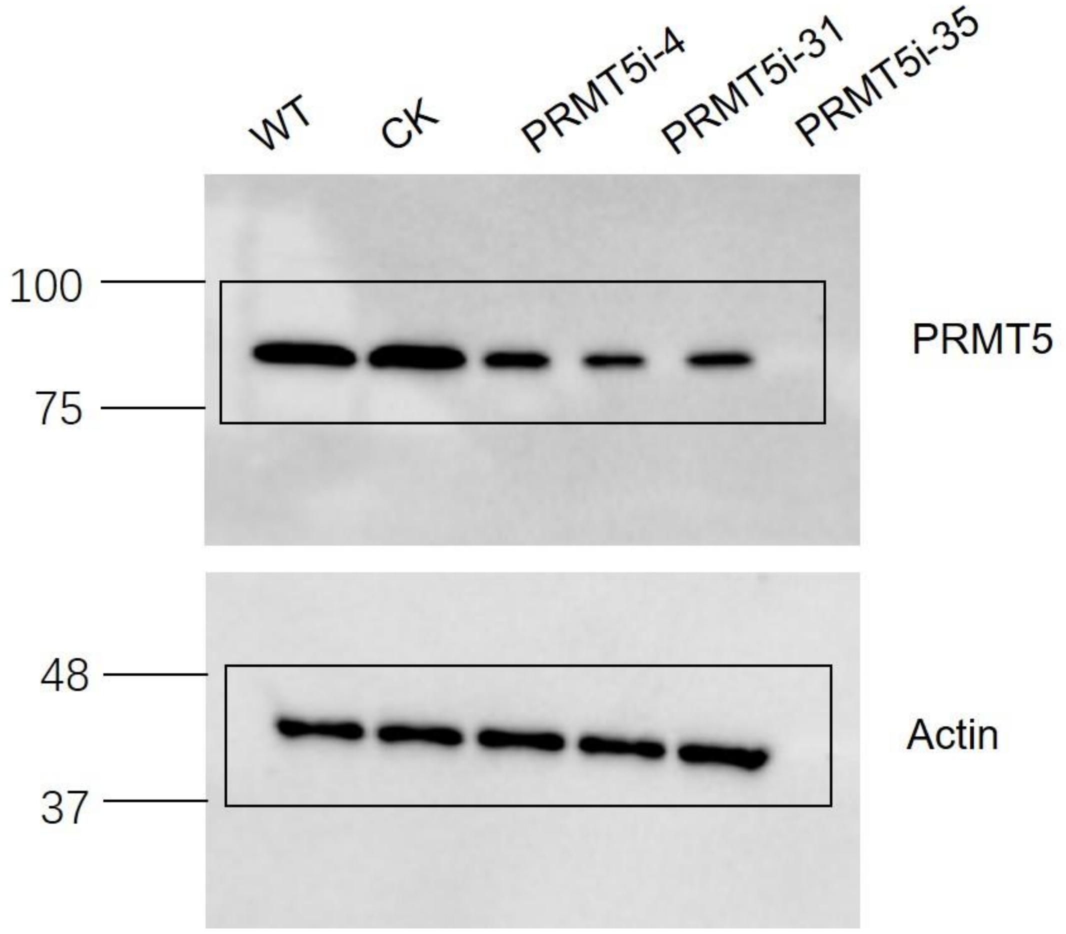

Supplementary Figure 4 Uncropped Western blots.

Source data for Figure 2a

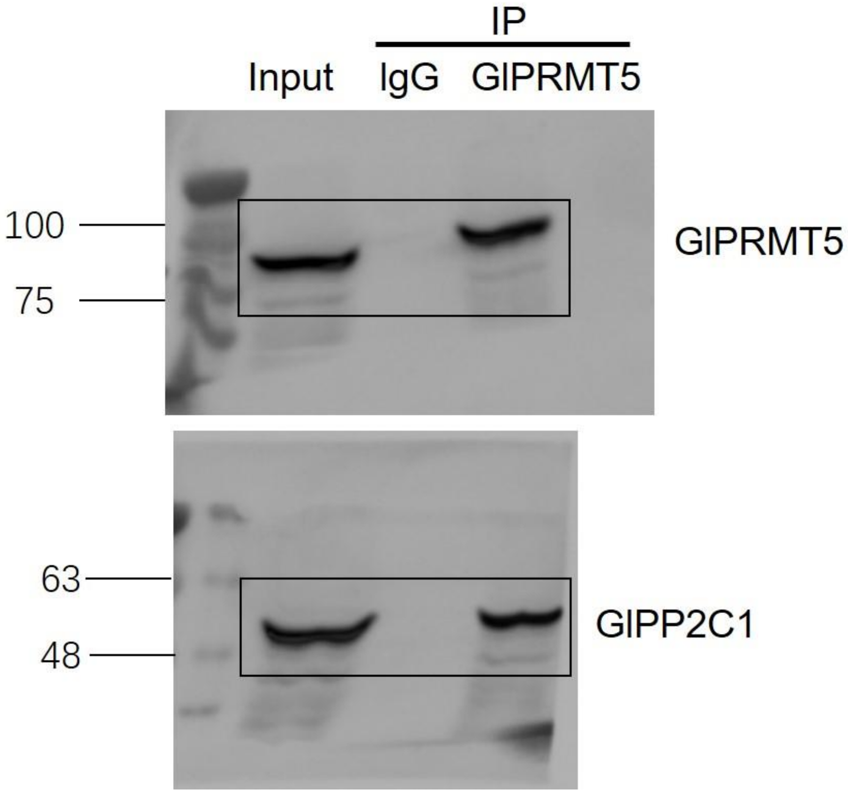

Supplementary Figure 5 Uncropped Western blots.

Source data for Figure 5a

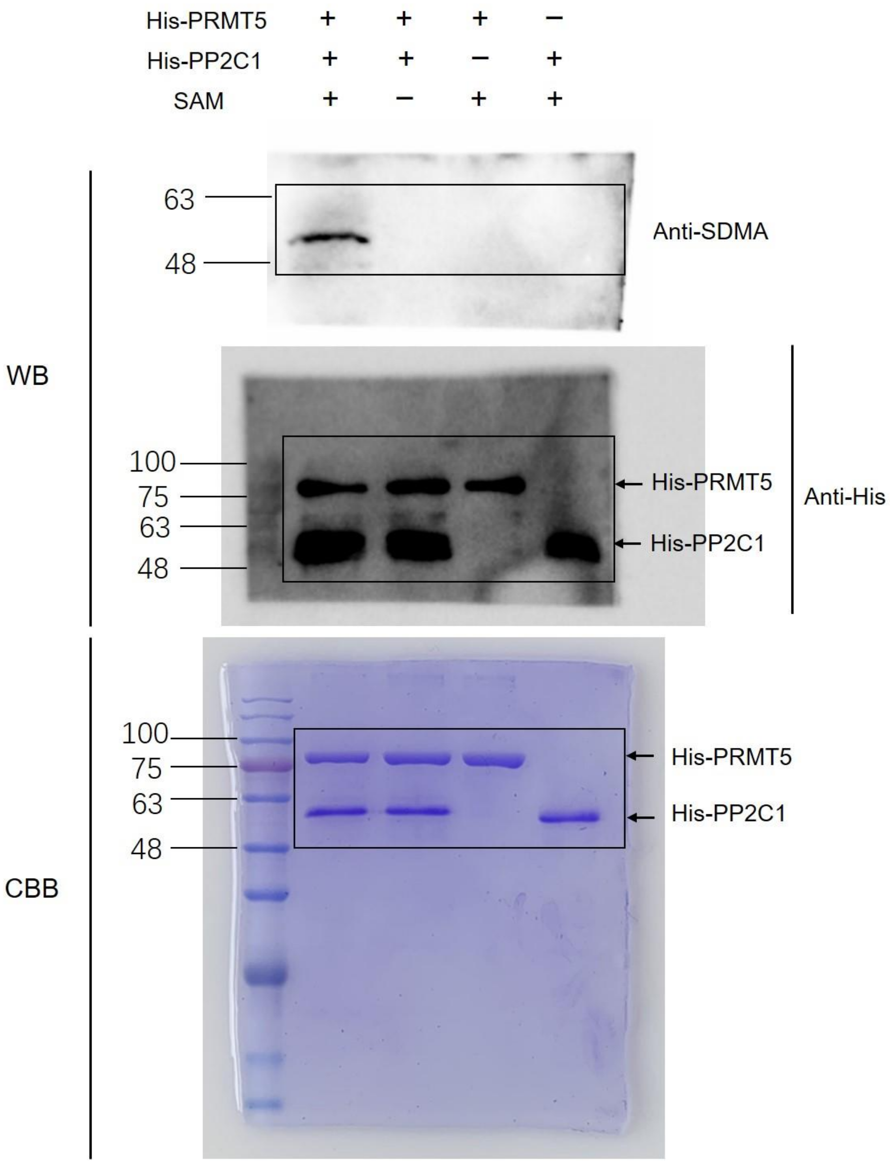

Supplementary Figure 6 Uncropped Western blots and gel.

Source data for Figure 6a

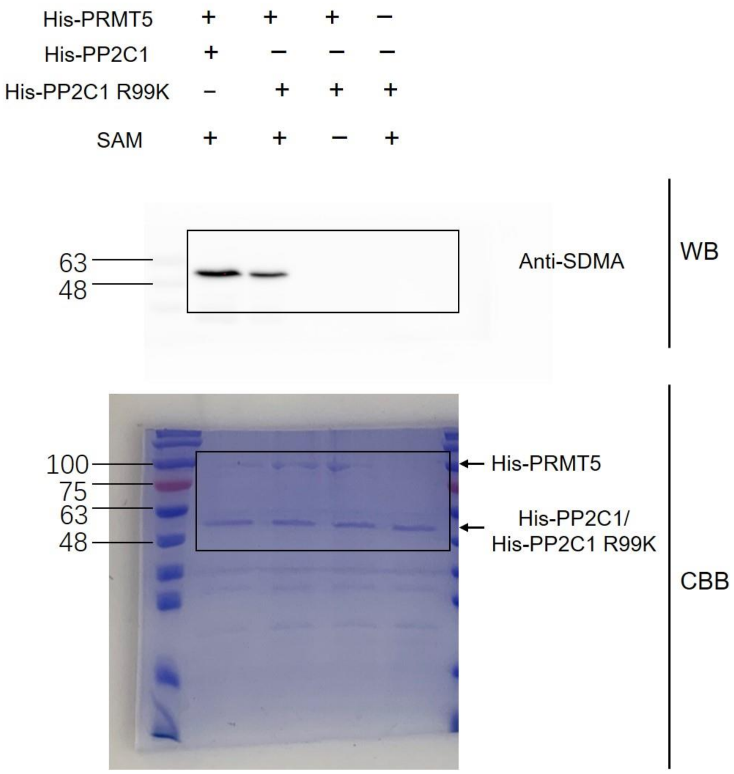

Source data for Figure 6b

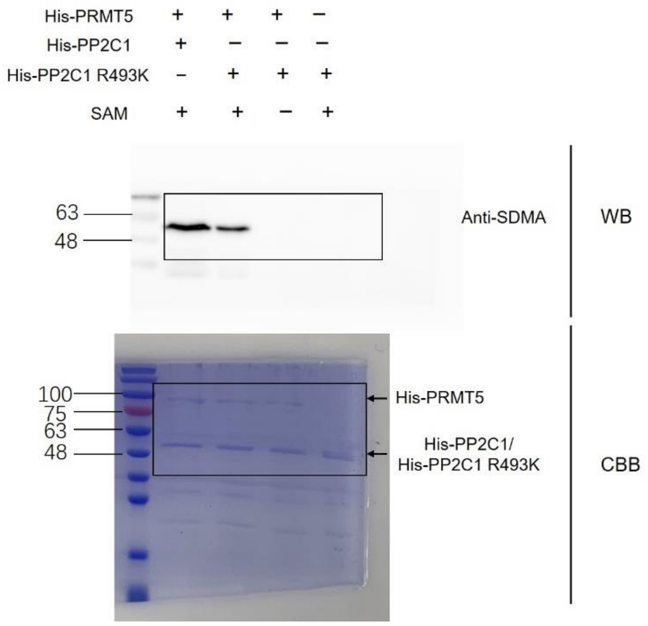

Source data for Figure 6c

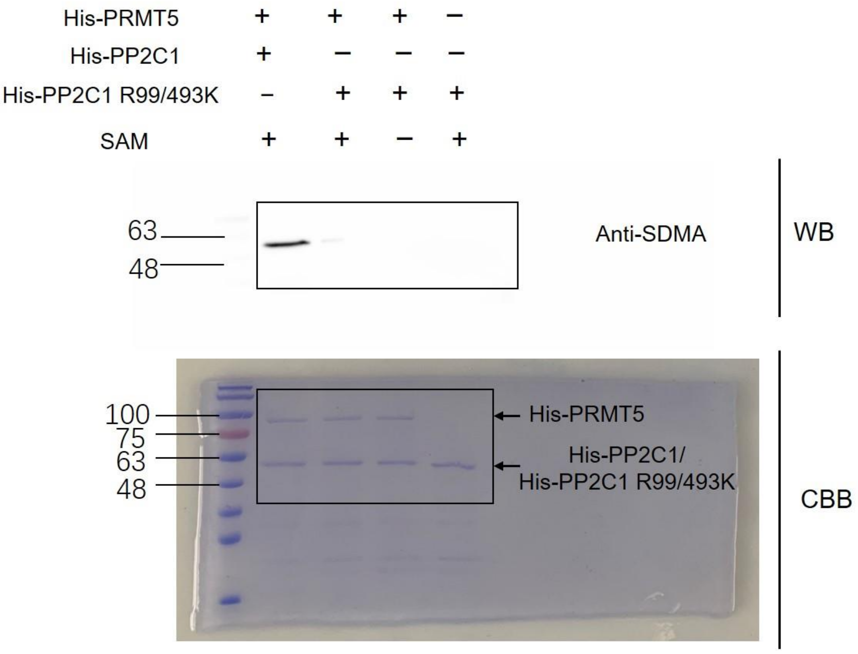

Supplementary Figure 7 Uncropped Western blots and gel.

Source data for Supplementary Figure 1b

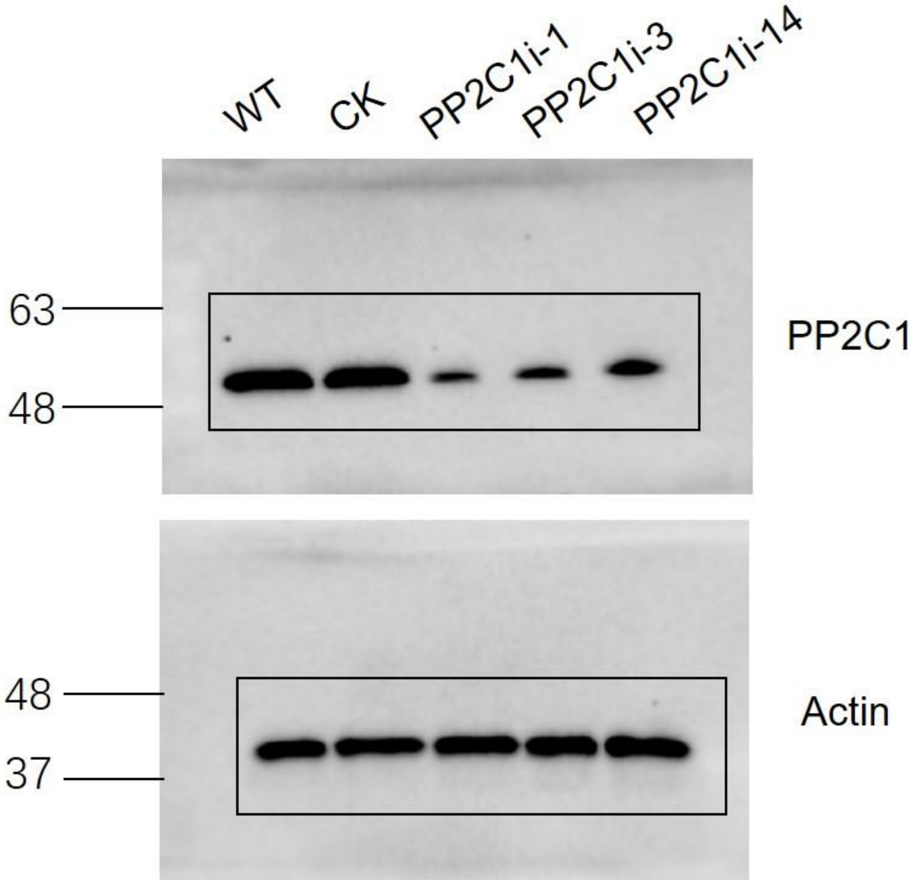

Source data for Supplementary Figure 1e

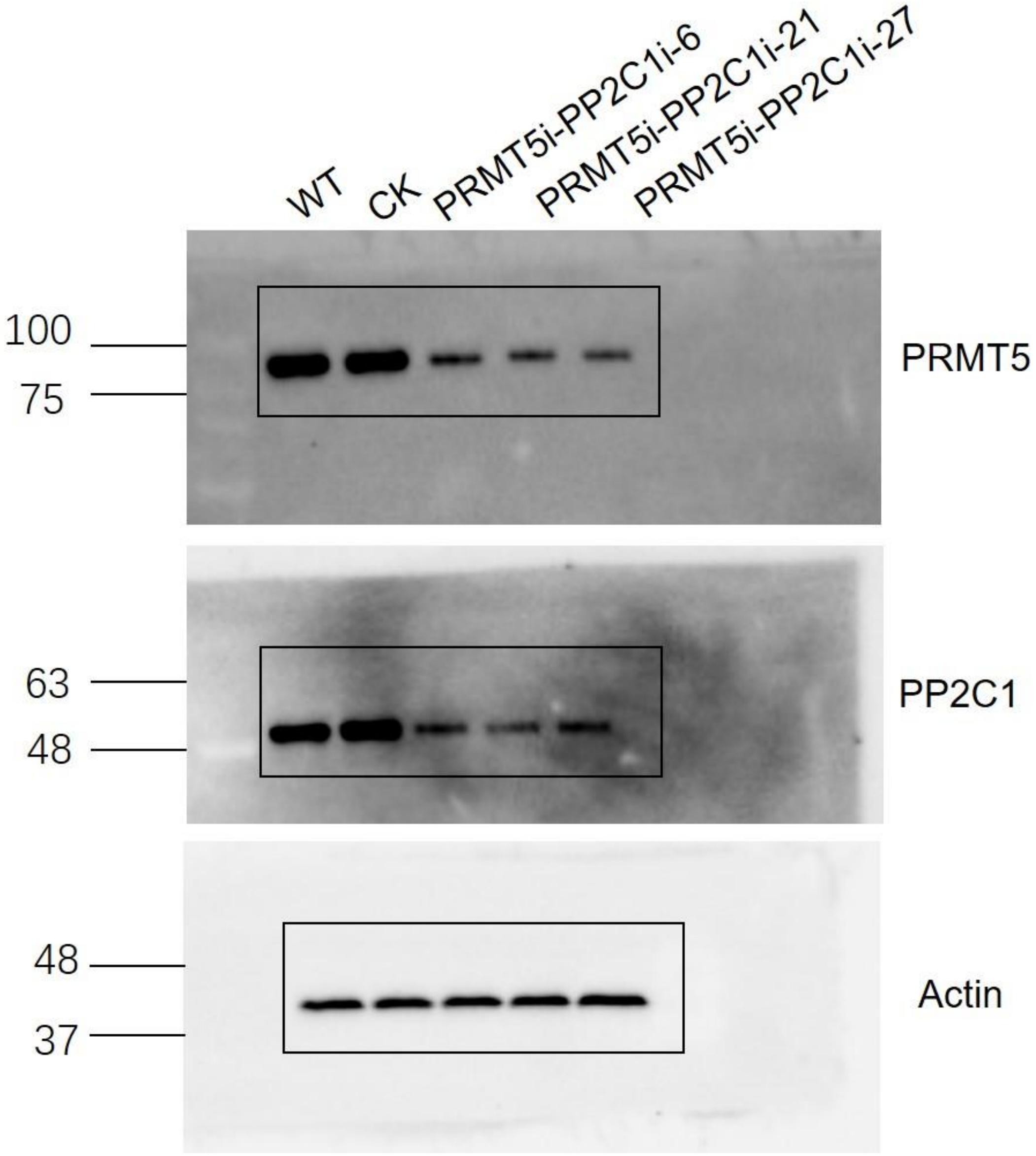

Supplementary Figure 8 Uncropped Western blots.

Source data for Supplementary Figure 3

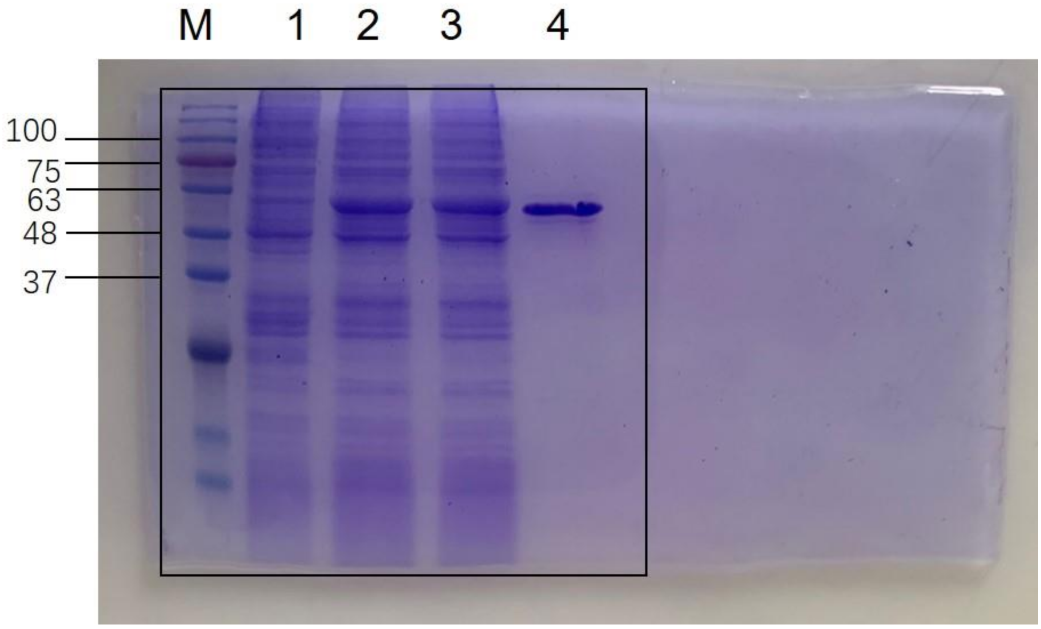

Supplementary Figure 9 Uncropped gel.

Supplementary Table 1. *Gl*PRMT5-interacting proteins identified by mass spectrometry

Accession	Coverage	Peptides	PSMs	Unique Peptides	Score	Peptides
GL22901-R1_1	51	16	24	16	91.87	16
GL30114-R1_1	15	5	6	5	19.51	5
GL29943-R1_1	7	6	8	6	17.36	6
GL22189-R1_1	7	2	3	2	10.7	2
GL24433-R1_1	10	3	4	3	10.4	3
GL18770-R1_1	19	3	3	3	8.47	3
GL30348-R1_1	9	2	3	2	7.76	2
GL24280-R1_1	2	1	2	1	6.65	1
GL30863-R1_1	3	1	2	1	6.22	1
GL20810-R1_1	3	2	2	2	5.68	2
GL23493-R1_1	16	2	2	2	5.23	2
GL28232-R1_1	4	2	2	2	5.04	2
GL24652-R1_1	2	1	2	1	4.9	1
GL21313-R1_1	7	2	2	2	4.64	2
GL29506-R1_1	2	1	2	1	4.56	1
GL22212-R1_1	5	2	2	2	4.24	2
GL26574-R1_1	4	2	2	2	4.23	2
GL25739-R1_1	3	2	2	2	4.11	2
GL22266-R1_1	14	1	1	1	3.88	1
GL30210-R1_1	6	1	2	1	3.68	1
GL22551-R1_1	8	1	1	1	3.65	1
GL21371-R1_1	2	1	1	1	3.62	1
GL24469-R2_1	3	1	1	1	3.54	1
GL19962-R1_1	6	1	1	1	3.53	1
GL30282-R1_1	4	1	1	1	3.53	1
GL21766-R1_1	5	1	1	1	3.44	1
GL26466-R1_1	3	1	1	1	3.42	1
GL19643-R1_1	20	1	1	1	3.21	1
GL19608-R1_1	4	1	1	1	3.14	1
GL20820-R1_1	4	1	1	1	3.13	1
GL20921-R1_1	10	1	1	1	3.1	1
GL21859-R1_1	2	1	1	1	3.1	1
GL29966-R1_1	11	1	1	1	3.1	1
GL22047-R1_1	9	1	1	1	3.1	1
GL18639-R1_1	3	1	1	1	3.06	1
GL15029-R1_1	20	1	1	1	3.05	1
GL24076-R1_1	5	1	1	1	3.04	1
GL19371-R1_1	3	1	1	1	3.03	1
GL28059-R1_1	5	1	1	1	2.94	1
GL22715-R1_1	3	1	1	1	2.82	1

Accession	Coverage	Peptides	PSMs	Unique Peptides	Score	Peptides
**GL15611-R1_1**	4	1	1	1	2.74	1
**GL24834-R1_1**	2	1	1	1	2.76	1
**GL17052-R1_1**	5	1	1	1	2.75	1
**GL21961-R1_1**	3	1	1	1	2.72	1
**GL16236-R1_1**	2	1	1	1	2.67	1
**GL25123-R1_1**	5	1	1	1	2.66	1
**GL19674-R1_1**	2	1	1	1	2.65	1
**GL27850-R1_1**	12	1	1	1	2.64	1
**GL20660-R1_1**	6	1	1	1	2.64	1
**GL22707-R1_1**	2	1	1	1	2.63	1
**GL21618-R1_1**	6	1	1	1	2.63	1
**GL24784-R1_1**	7	1	1	1	2.62	1
**GL17531-R1_1**	4	1	1	1	2.62	1
**GL17923-R1_1**	8	1	1	1	2.61	1
**GL17506-R1_1**	11	1	1	1	2.61	1
**GL30776-R1_1**	5	1	1	1	2.6	1
**GL23780-R1_1**	4	1	1	1	2.6	1
**GL30213-R1_1**	5	1	1	1	2.59	1
**GL24083-R1_1**	4	1	1	1	2.56	1
**GL23971-R1_1**	3	1	1	1	2.54	1
**GL20287-R1_1**	7	1	1	1	2.49	1
**GL24672-R1_1**	3	1	1	1	2.49	1
**GL18123-R1_1**	6	1	1	1	2.48	1
**GL25744-R1_1**	6	1	1	1	2.47	1
**GL15392-R1_1**	4	1	1	1	2.38	1
**GL30148-R1_1**	1	1	1	1	2.37	1
**GL18315-R1_1**	5	1	1	1	2.35	1
**GL16746-R1_1**	5	1	1	1	2.32	1
**GL30680-R1_1**	2	1	1	1	2.3	1
**GL29305-R1_1**	1	1	1	1	2.27	1
**GL24238-R1_1**	1	1	1	1	2.26	1
**GL23055-R1_1**	1	1	1	1	2.18	1
**GL20175-R1_1**	9	1	1	1	2.17	1
**GL29728-R1_1**	3	1	1	1	2.11	1
**GL16996-R1_1**	6	1	1	1	2.09	1
**GL30579-R1_1**	4	1	1	1	2.07	1
**GL22245-R1_1**	2	1	1	1	2.05	1
**GL24246-R1_1**	2	1	1	1	2.04	1
**GL21501-R1_1**	1	1	1	1	2.04	1
**GL30254-R1_1**	1	1	1	1	2.01	1
**GL21857-R1_1**	2	1	1	1	2	1
**GL21672-R1_1**	2	1	1	1	1.98	1
**GL19040-R1_1**	3	1	1	1	1.98	1

Accession	Coverage	Peptides	PSMs	Unique Peptides	Score	Peptides
**GL18813-R1_1**	1	1	1	1	1.97	1
**GL27854-R1_1**	3	1	1	1	1.96	1
**GL21834-R1_1**	2	1	1	1	1.95	1
**GL29535-R1_1**	1	1	1	1	1.95	1
**GL29900-R1_1**	1	1	1	1	1.95	1
**GL19167-R1_1**	10	1	1	1	0	1
**GL21533-R1_1**	2	1	1	1	0	1
**GL30943-R1_1**	5	1	1	1	0	1
**GL21970-R1_1**	5	1	1	1	0	1
**GL18120-R1_1**	2	1	1	1	0	1
**GL25223-R1_1**	4	1	1	1	0	1
**GL29329-R1_1**	2	1	1	1	0	1
**GL24019-R1_1**	10	1	1	1	0	1
**GL28205-R1_1**	10	1	1	1	0	1

Supplementary Table 2. Oligonucleotide primers used

Primer	Sequence (5′to 3′)	Description
PP2C1i-F	GGGGTACACTCGCATTCCCGCTT	Used to get the silencing fragment of the *GlPP2C1* gene
PP2C1i-R	GACTAGTTCGCTCGTCAGGCTTT	
RT-PP2C1-F	GGGAAAGCCTGACGAGCGA	Used to detect the *GlPP2C1* expression
RT-PP2C1-R	GCCATCCACACGGGCAACG	
RT-18S-F	TCGAGTTCTGACTGGGTTGT	Used to detect the 18S rRNA expression
RT-18S-R	TCCGTTGCTGAAAGTTGTAT	
PRMT5i-F	GATCGGTACCTCTGGATGGCGGGATG	Used to get the silencing fragment of the *GlPRMT5* gene
PRMT5i-R	GATCACTAGTGCGGGAACCAACTAAGCA	
RT-PRMT5-F	CCTTCATTCGGGACATCTT	Used to detect the *GlPRMT5* expression
RT-PRMT5-R	TCGTCGCAGCCTTCACA	
